# Supplementary material for: Modelling ribosome kinetics and translational control on dynamic mRNA
Source: PLoS Comput Biol. 2023 Jan 23;19(1):e1010870. doi: 10.1371/journal.pcbi.1010870 (PMC9894550; doi:10.1371/journal.pcbi.1010870)
Supplement: S1 Text — Fig A: Tree representation of the secondary structure of the bacteriophage MS2 coat gene. Secondary structural elements (hairpins and multi-loop helices) are labelled 1–15, with 0 used to label the exterior loop. The tree representation of the secondary structure is shown in the upper left, with each node of the tree representing one of the helix elements, i.e. a hairpin or multi-loop helix. Black arrows indicate a linked list pointing to the 5’ and 3’ neighbours of each structural element. Green arrows point to leaf nodes, the 5’ most helix element in a multi-loop, while red arrows point to root nodes, i.e. the multi-loop helix which closes the multi-loop that the node is apart of. Fig B: Tree representation of the secondary structure of the bacteriophage MS2 coat gene with bound ribosome. Secondary structural elements (hairpins and multi-loop helices) and ribosomes bound to the mRNA are labelled 1–15, with 0 used to label the exterior loop. Yellow nucleotides indicate the footprint of the 70S ribosome while purple nucleotides indicate the location of the ribosome P-site. The tree representation of the secondary structure is shown in the upper left, with each node of the tree representing either a helix element or bound ribosome. Links between nodes (black,green and red arrows) follow the same rules as in Fig A. Fig C: Example of how a local hairpin RNA folding transition is constructed. The bacteriophage MS2 coat gene and its tree representation are given with each node in the tree representing either a helix or ribosome. The yellow shaded nucleotides give the ribosome footprint on the mRNA while the purple nucleotides denote the location of the P-site. The blue nucleotides colour the nucleotides which make up the window fragment. This window is extracted and the lowest energy RNA fold computed. The window fragment is replaced with the lowest energy fold to construct the new RNA fold, and the folding transition rate (kF) is computed using a breadth-first-search ba [file pcbi.1010870.s001.pdf]

S1 Text

Parameters for Prokaryote Ribosome Kinetics Model

Here I detail the kinetic and protein concentrations parameters used in the translation model and give details on how an approximate transcriptome for *E. coli* K12 strain MG1655 was constructed. Kinetic parameters used in this study follow those developed in my previous paper (1) which examined an *in vivo* stochastic model for the simulation of ribosome kinetics in a whole *E. coli* cell. The protein concentrations used here are identical to those used in (1), with the exception of tRNA abundances and release factors 1 and 2, which must be optimised to match the codon bias present in MG1655. This is discussed below followed by information on how an approximate transcriptome of *E. coli* K12 strain MG1655 mRNAs was constructed for different bacterial growth rates.

Adjustment of tRNA abundances for Ecoli K12 strain MG1655. In previous work (1) I demonstrated that the tRNA measurements of Dong and colleagues (2) resulted in poor translational efficiency and increased stalling of translating ribosomes when mRNAs from E. coli K12 were used for the transcriptome. I hypothesised this to be due to small inaccuracies in the *in vivo* tRNA abundances measured by Dong et al. which do not match precisely with the codon bias found in E. coli K12 mRNAs. Although there are alternative explanations that could account for the decreased translational efficiency (e.g. a missing kinetic reaction pathway in the model, or effects from amino-acid tRNA synthetases), I demonstrate here that altering the tRNA abundances used in the translation model so that they roughly match the E coli K12 mRNA codon bias is sufficient to restore the translational speeds to levels expected from experiment (3, 4).

The tRNA abundance profile used for simulation with Ecoli K12 mRNAs (strain MG1655 - uniprot accession code U00096) was adjusted as follows. First, the codon frequency was estimated using the genes identified in the uniprot database. Excluding the three stop codons, this gave a codon frequency for each codon f_c. The f_c values where normalised such that,

$$\sum_c f_c = 1,$$

where the sum is over all sense codons. Next, codons were separated into two groups, those that are decoded by a single tRNA species (group 1 codons) and those that are decoded by multiple tRNAs (group 2 codons). For example, tRNA^{Lys} is the only tRNA which can decode codons AAA and AAG, while the codon CCG can be decoded by two tRNAs, tRNA^{Pro3} and tRNA^{Pro1}. Any tRNAs which only decode group 1 codons were immediately assigned an abundance using the total number of tRNAs expected in the cell, N_T, multiplied by the sum of the frequencies of the codons they decode. For example, tRNA^{Lys} decodes AAA and AAG codons and hence, the expected number of tRNA^{Lys} in the cell (N_{Lys}) is given by

$$N_{Lys} = (f_{AAA} + f_{AAG})N_T$$

At growth rate $\mu = 0.7$, Bremer and Dennis (3) expect there to be on the order of $N_T = 72$k tRNAs in an average *E. col* cell. Thus for the above example with tRNA^{Lys}, $N_{Lys} = 3065$ for $\mu = 0.7$ (c.f. S1 Table).

For tRNAs that decode a codon from group 2, there exists an inter-dependence between multiple tRNAs which does not allow for a simple assignment of tRNA abundance based on codon frequency like with the tRNAs which decode group 1 codons. The examples of tRNA^{Pro1}, tRNA^{Pro2} and tRNA^{Pro3} can be used as an illustration of the issue. Since these three tRNAs only decode the four codons CCx, with x=A,G,C,U, it can be expected that

$$N_{Pro1} + N_{Pro2} + N_{Pro3} = (f_{CCA} + f_{CCC} + f_{CCG} + f_{CCU})N_T.$$

However, since tRNA^{Pro3} and tRNA^{Pro1} both decode the CCG codon, and tRNA^{Pro3} and tRNA^{Pro2} both decode the CCU codon, there are multiple ways in which this constraint can be satisfied. There are six additional sets of tRNAs having this same issue, and thus seven abundance constraints can be formulated as

$$\begin{aligned}
N_{Pro1} + N_{Pro2} + N_{Pro3} &= (f_{CCA} + f_{CCC} + f_{CCG} + f_{CCU})N_T, \\
N_{Thr1+3} + N_{Thr2} + N_{Thr4} &= (f_{ACA} + f_{ACC} + f_{ACG} + f_{ACU})N_T, \\
N_{Gly1} + N_{Gly2} + N_{Gly3} &= (f_{GGA} + f_{GGC} + f_{GGG} + f_{GGU})N_T, \\
N_{Ser1} + N_{Ser2} + N_{Ser5} &= (f_{UCA} + f_{UCC} + f_{UCG} + f_{UCU})N_T, \\
N_{Val1} + N_{Val2} &= (f_{GUA} + f_{GUC} + f_{GUG} + f_{GUU})N_T, \\
N_{Leu1} + N_{Leu3} &= (f_{CUA} + f_{CUG})N_T, \\
N_{Leu4} + N_{Leu5} &= (f_{UUA} + f_{UUG})N_T.
\end{aligned}$$

The right hand sides of the constraint equations are constants, and thus they can be thought of as either a constraint to the unit sphere (in the case of three coupled tRNAs) or a constraint to the unit circle (for the case of two coupled tRNAs). Thus, the first four constraints can be encoded using two parameters and the last three encoded using one parameter. This gives 11 parameters in total which can be varied to cover the full solution space. To identify a solution to these constraint equations which maximises the translational speed, I employed a steepest accents gradient search on the 11 parameters, which can be encoded into a vector $x_i^{(n)}$ where n gives the current step number in the steepest accent search, and i ranges over the 11 parameters. Let $C_p(\mathbf{x})$ denote the average ribosome translational speed for the given parameters x_i. Then the components of the gradient g_i with $\mathbf{g} = \nabla C_p(\mathbf{x})$ can be calculated numerically using

$$g_i = \frac{\partial C_p(\mathbf{x})}{\partial x_i} \approx \frac{C_p(\mathbf{x} + h\mathbf{e_i}) - C_p(\mathbf{x})}{h}$$

where h is real number and $\mathbf{e}_i$ is the 11-d unit vector containing a 1 in the ith position.

Thus, the 11 components of the gradient can be computed using 12 simulations of translation at appropriate coordinates $\mathbf{x}$ in the parameter space. I use a growth rate of $\mu = 0.7$ along with the codon frequencies f_c and total tRNAs, $N_T = 72$k, estimated by Bremer and Dennis (3) and perform a maximisation of $C_p(\mathbf{x})$ using a steepest accents gradient search. The resulting tRNA abundances at $\mu = 0.7$ are given in S1 Table, with tRNAs extrapolated to higher growth rates. The average translational speed for the tRNA abundances in Table 1 was found to be $C_p = 14.5$ ($\mu = 0.7$), $C_p = 16.0$ ($\mu = 1.06$), and $C_p = 18.3$ ($\mu = 2.5$). These average translational speeds, although slightly lower then expected by Bremer and Dennis (3) are in line with other experimental measurements done on the average translational speeds of the β-galactosidase mRNA in K12 strain MG1655 (4). I calculate average translational speeds of the LacZ mRNA as, $C_p = 14.7$ ($\mu = 0.7$), $C_p = 16.9$ ($\mu = 1.06$), and $C_p = 19.0$ ($\mu = 2.5$) closely following experimental measurements (4).

Estimates of the misreading rate by tRNA^{Lys} of near-cognate codons were also computed in the translation simulations (S2 Table) and compared with experiment (5). The misreading rates of near-cognate codons AGA, AGG, AAU, AAC, AUG, CAA, and CAG were essentially off by a uniform factor of 3 (c.f. S2 Table), suggesting that relative ratios of tRNAs are a good approximation to reproduce tRNA^{Lys} misreading events measured by experiment. Adjustments to the near-cognate elongation kinetic rates would be likely needed to bridge the gap between theory and experiment. The concentration of release factors RF1 and RF2 were adjusted to match the experimental tRNA^{Lys} misreading rates at stop codons UAA and UAG, as the previous concentrations used in (1) were slightly too low. The optimised RF1 and RF2 amounts are 0.016 and 0.060 per ribosome, which correspond to $n_{rf1} = 240$ copies of RF1 and $n_{rf2} = 903$ copies of RF2 in the *E. coli* cell at a growth rate $\mu = 1.06$.

Transcriptome Generation for Ecoli K12 strain MG1655. An approximate transcriptome for *E. coli* K12 strain MG1655 at different growth rates $\mu = 0.7, 1.06$, and 2.5 was created as follows. The genome of *E. coli* K12 (uniprot id U00096) and its predicted proteome (uniprot UP000000625) was used to identify the open reading frames for each protein in the predicted proteome. These ORFs were then used to extract the mRNA sequence corresponding to the reading frame. A total of 4365 ORFs were identified and a mRNA sequence was constructed for each. This created a database of truncated mRNAs lacking 5' and 3' UTRs which could subsequently be used to construct approximated transcriptomes.

To construct a transcriptome I follow my previous procedure (1), i.e. individual mRNAs from the *E. coli* K12 database corresponding to a reading frame for a single gene are selected at random, and the number of copies for the mRNA is assigned into either a high (probability 5%), intermediate (probability 35%), or low (probability 60%) expression category. Copy numbers (k) for each mRNA are then determined by sampling from either a Poisson distribution, $p(k) = e^{-\lambda}\lambda^k/k!$, for the high expression ($\lambda = 6.8$), or a geometric distribution, $p(k) = (1-\lambda)^k\lambda$, for the intermediate ($\lambda = 0.58$) and low ($\lambda = 0.93$) expression categories. Genes and their corresponding mRNA sequences are selected, one at a time, until a total nucleotide content and/or total number of mRNAs is achieved. For growth rate $\mu = 0.7$ a total nucleotide content of 0.56M nucleotides was used, resulting in a total of 650 mRNAs. Similarly, at growth rates $\mu = 1.06$ and $\mu = 2.5$, a total nucleotide content of 1.16M and 4.74M nucleotides, respectively, was used resulting in 1257 and 5151 mRNAs in the respective transcriptomes.

Stochastic Model of Co-Translational mRNA Folding

Here I detail the design of a stochastic co-translational mRNA folding model which has been developed in such a way that it can be implemented in tandem with the *in vivo* model of prokaryote ribosome kinetics discussed in (1). There are several key issues which must be considered when implementing a stochastic co-translational mRNA folding model; (1) the choice of possible folding reactions (i.e. kinetic transitions to alternative structures) and their likelihood to reproduce a reasonably accurate picture of folding kinetics, (2) the computational time required to calculate the reaction rates for these folding reactions, and (3) the implementation of a data structure to hold both the current mRNA fold as well as information about ribosomes and proteins bound to it. On the one hand, folding reactions could be chosen to be at single base-pair resolution, i.e. as implemented by Kinfold or Kfold (6, 7). In this Kinfold/Kfold view, a folding reaction is a transition between two structures differing by a single base-pair. However, this detailed view of the folding kinetics is both time consuming computationally and also likely unnecessary, as the time frame for most local hairpin folding reactions are in the millisecond range, while the movement of the ribosome is in the time frame of > 50 ms. Thus, in most instances, the mRNA is likely to adopt its lowest energy folding configuration (as long as it is kinetically accessible from its current structure) within the time frame of ribosomal movement. Likewise, protein binding to structures in the mRNA is also likely to be slower then local hairpin refolding. One can estimate that protein would bind to mRNA in a time frame of $\tau = 1/k_f[P]$ where k_f is the binding rate and $[P]$ is the concentration of protein. For the most abundant protein in the cell (Ef-Tu), concentrations range between 0.1-0.2 mM (3). This gives for the most abundant protein in the cell a binding time on the order of 5 ms. Hence, a course-grained picture of mRNA folding in which a folding reaction is a transition to a new fold where multiple base-pair additions and deletions have occurred should be a good approximation to the folding kinetics here.

To design a course-grained model of mRNA folding kinetics, I follow the ideas of the Kinwalker program (8), which is a coarse-grained model for studying the kinetics of RNA transcriptional folding, but make a few key changes to allow the model to be computationally efficient when implemented within the prokaryote ribosome model. The next sections detail these changes, along with a description of (1) the data structure used to hold mRNA structural information, (2) how a set of new

mRNA folding states that can be transitioned to from the current mRNA fold are determined, and (3) how the transition rates to these new mRNA states are computed.

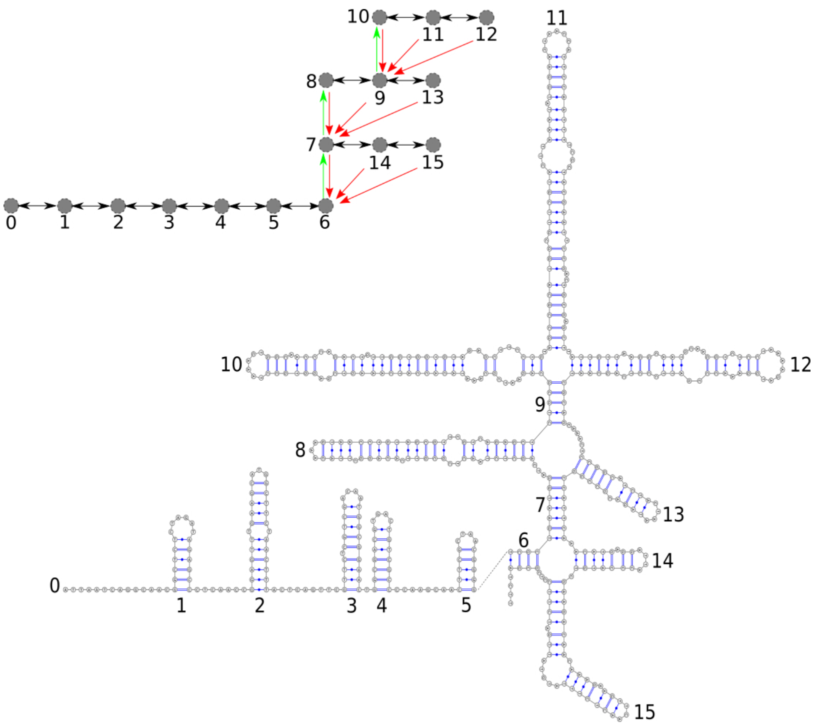

Figure A. Tree representation of the secondary structure of the bacteriophage MS2 coat gene. Secondary structural elements (hairpins and multi-loop helices) are labelled 1-15, with 0 used to label the exterior loop. The tree representation of the secondary structure is shown in the upper left, with each node of the tree representing one of the helix elements, i.e. a hairpin or multi-loop helix. Black arrows indicate a linked list pointing to the 5' and 3' neighbours of each structural element. Green arrows point to leaf nodes, the 5' most helix element in a multi-loop, while red arrows point to root nodes, i.e. the multi-loop helix which closes the multi-loop that the node is apart of.

Tree Representation of mRNA Structure. The secondary structure of an mRNA with N nucleotides can easily be stored in a single array of size N where `ibsp(i)=j` if nucleotide i is base-paired to nucleotide j, and `ibsp(i)=0` if nucleotide i is single-stranded. However, for large mRNAs, this data structure does not efficiently enable the search for local helix structures such as hairpins and multi-loop helices, nor does it allow for easy identification of single-stranded bases that are ribosome bound, or helix elements that are protein bound. For the purposes of the ribosome and its interaction with the mRNA, the important structural features that need to be accounted for are; (1) hairpin helices, (2) multi-loop helices, (3) stretches of un-interrupted single-stranded nucleotides between helix elements. Moreover, a good data structure should allow for the easy search and identification of these three structure types. Therefore, I use a tree representation to store a coarse-grained picture of the mRNA structural elements, along with the `ibsp()` array to store the exact base-pairing information for the mRNA. Fig A shows a diagram of the tree representation for the secondary structure of the bacteriophage MS2 coat gene. Here, the secondary structural elements are labelled 1-15, with 0 used as a special label for the 5' end of the exterior loop. These structural elements can be abstracted to "nodes" (top left corner of Fig A) on a tree, with arrows indicating the linked list pointing to the 5' and 3' neighbouring structural elements (black arrows). Nodes with a green arrow leaving the node indicate that the node is a multi-loop helix element, and the arrow points to the first 5' structural element in the multi-loop. Likewise, red arrows leaving a node indicate that the node is a structural element that is part of a multi-loop, and the arrow points to the helix which closes the multi-loop. I will refer to the nodes that green arrows point to as "leaf nodes" and the nodes that red arrows point to as "root nodes", akin to leafs and roots in a tree.

The tree data structure tracks the following information for each node in the tree; (1) the position of the first and last base-pair in the helix that the node refers to, (2) the leaf and root nodes, and (3) the 5' and 3' neighbouring nodes. This allows for structural elements in the mRNA to be quickly traversed via the links, and for hairpins and multi-loop helices to be easily distinguished since nodes corresponding to multi-loop helices are the only nodes having a non-zero leaf node. When a ribosome is present on the mRNA, either as the 30S:PIC (30S pre-initiation complex) or an elongating 70S ribosome, then the tree structure accounts for this by marking the ribosome as a special node. Fig B illustrates how the tree structure changes when a translating ribosome is bound to the start region of the bacteriophage MS2 coat gene. The purple nucleotides indicate the position of the ribosome P-site, while the yellow nucleotides give the ribosomal footprint. One can notice that the hairpins corresponding to nodes 3, 4, and 5 are altered when compared to those in Fig A, showing how my model predicts new mRNA folds in response to the ribosome position on the mRNA.

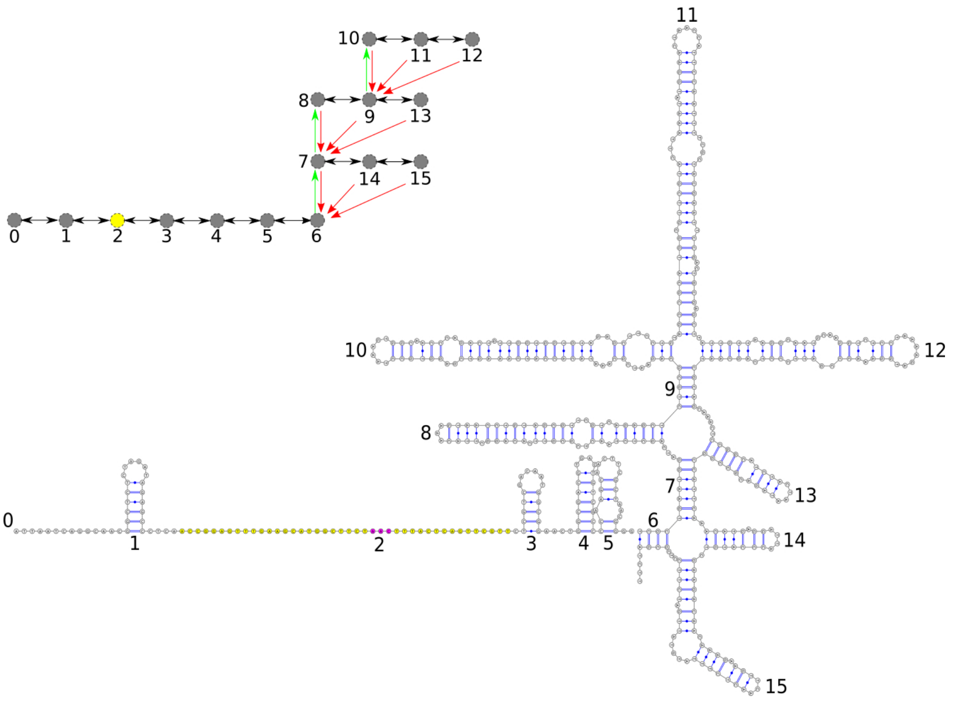

Figure B. Tree representation of the secondary structure of the bacteriophage MS2 coat gene with bound ribosome. Secondary structural elements (hairpins and multi-loop helices) and ribosomes bound to the mRNA are labelled 1-15, with 0 used to label the exterior loop. Yellow nucleotides indicate the footprint of the 70S ribosome while purple nucleotides indicate the location of the ribosome P-site. The tree representation of the secondary structure is shown in the upper left, with each node of the tree representing either a helix element or bound ribosome. Links between nodes (black,green and red arrows) follow the same rules as in Fig A.

Determination of a set of mRNA structural transitions. For the coarse-grained modelling of the kinetics of mRNA folding in response to interactions with proteins and ribosomes, it is necessary to determine a sub-set of secondary structures for the mRNA which approximates the complete set of mRNA structures that are kinetically accessible from its current structure. In the coarse-grained modelling of RNA folding kinetics, these structures will differ from the current structure by many base-pairs unlike with single base-pair resolution kinetic models (e.g. KFOLD, Kinfold) where they differ by at most one base-pair. In essence, coarse-grained kinetic folding models rely on identifying RNA structural states which have re-structured a large section of the RNA when compared to the current fold.

In previous work on coarse-grained kinetic folding models, Flamm and colleagues developed the program Barriers (9) which constructs a set of RNA structural states that can be kinetically transitioned to by identifying local minima in the folding energy landscape in a neighbourhood around the current RNA structure. This can be considered the "gold-standard" approach as the Barriers algorithm essentially exhaustively searches the folding landscape for all neighbouring local minima. These local minima typically differ by many base-pair additions and deletions from the current structure and thus represent a more coarse-grained view of the folding landscape. However, this method is extremely computationally expensive, as the search for local minima scales exponentially with the size of the neighbourhood. This makes fast coarse-grained kinetic folding models using the Barriers algorithm computationally expensive.

Because of this issue, the Kinwalker program (8) was developed which uses local folding windows to determine the RNA structures that can be transitioned to as opposed to exhaustive searching of for local minima in the folding energy landscape. To construct an RNA structure that can be kinetically transitioned to, Kinwalker computes the minimum energy fold on a local window of nucleotides $[i, j]$ using standard dynamic programming algorithms for RNA folding. Kinwalker then considers the transition from the current RNA fold, to a new RNA fold where the fold on nucleotides $[i, j]$ has been replaced by the minimum energy fold. Kinwalker uses a specific procedure to choose appropriate windows $[i, j]$ such that conflicts between base-pairing upstream and downstream on the fragment $[i, j]$ do not occur. I have chosen to compute RNA transition states using a similar procedure of selecting local folding windows on sections of nucleotides $[i, j]$ and computing the minimum energy folds on these window fragments. However unlike Kinwalker, the locations of window fragments used in the ribosome model are based on the current locations of helices and ribosomes on the mRNA. This choice is made to ensure a computationally efficient kinetic co-translational folding model. Specifically, as the ribosome translocates down the mRNA, the list of potential RNA kinetic folding events must be updated in order to accommodate the new position of the ribosome. By using RNA folding windows determined by the neighbouring structural elements (i.e. up/down stream helices and ribosomes) only a few windows, and their corresponding minimum energy folds and kinetic rates, will need to be updated. Furthermore, I have separated the RNA folding transitions into two types; (1) those which involve the formation of local hairpins and (2) those that involve the formation of a multi-loop. This is necessary to ensure multi-loops do not form around ribosomes, which I have made a forbidden reaction in my model at this stage of development.

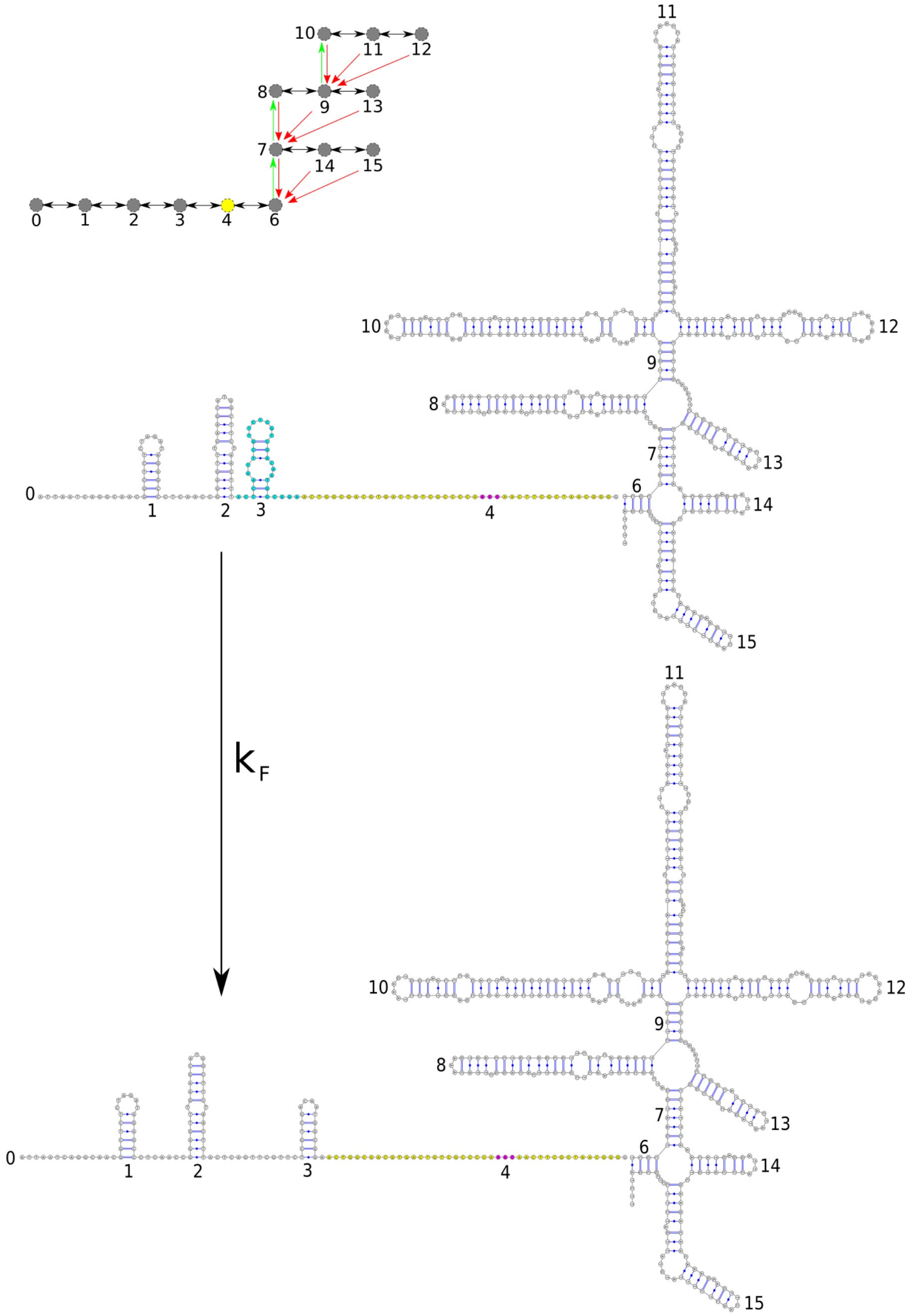

Figure C. Example of how a local hairpin RNA folding transition is constructed. The bacteriophage MS2 coat gene and its tree representation are given with each node in the tree representing either a helix or ribosome. The yellow shaded nucleotides give the ribosome footprint on the mRNA while the purple nucleotides denote the location of the P-site. The blue nucleotides colour the nucleotides which make up the window fragment. This window is extracted and the lowest energy RNA fold computed. The window fragment is replaced with the lowest energy fold to construct the new RNA fold, and the folding transition rate (k_F) is computed using a breadth-first-search barrier prediction algorithm.

Structural Transitions Involving Local Hairpin Formation. To illustrate how an mRNA structural transition involving local hairpin formation can be constructed using nucleotide window fragments $[i, j]$, I begin with an example of the mRNA from the bacteriophage MS2 coat gene which has a bound ribosome as shown in Fig C. In this example, the blue shaded region corresponds to a window fragment containing a single hairpin and all of the single-stranded bases which are 5' and 3' to the hairpin. The lowest energy RNA fold on this window fragment is then calculated using standard dynamical programming techniques for RNA folding. Replacement of the fold in this window with the lowest energy fold results in the new RNA structure below. The transition rate for the folding reaction, k_F, can be computed using a greedy barrier estimation technique. Details of this algorithm are discussed in the following section.

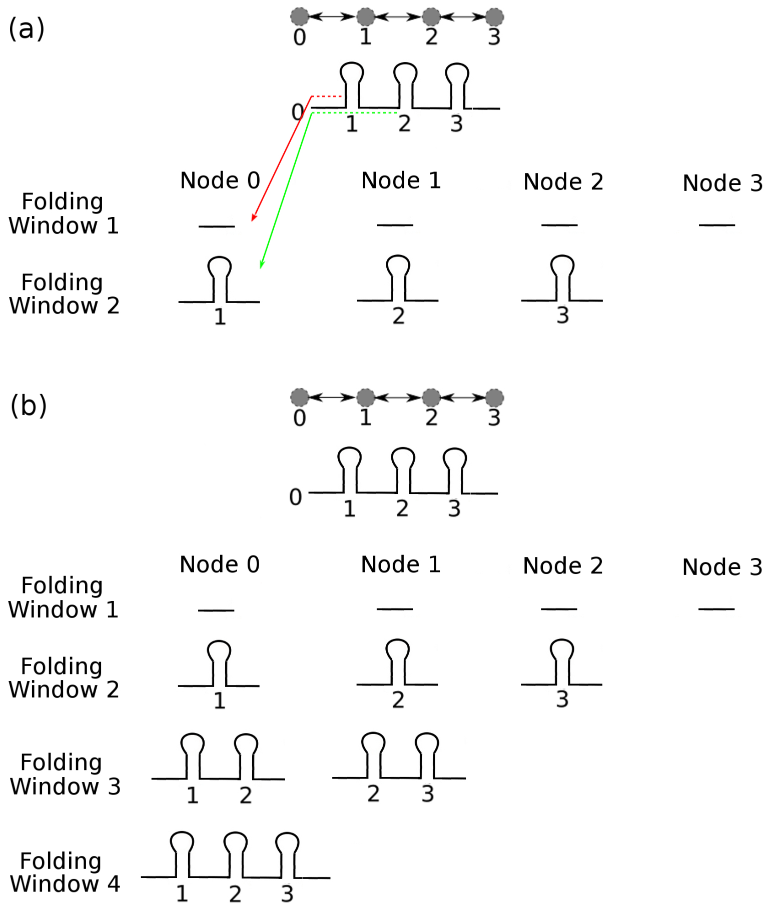

Figure D. Construction of folding windows. (a) Example of how folding windows are constructed for a simple RNA structure consisting of three hairpins and no multi-loops. Here $N_w = 2$ and two folding windows are constructed for each node in the tree. Red and green arrows depict how sections of the RNA are extracted for each window (example for node 0 shown). (b) Example of folding window construction on the same RNA structure as in (a), but with $N_w = 4$. For this setting and RNA fold, all possible window fragments will be considered.

In general, the procedure for constructing transitions which have local re-arrangements of the hairpins in the mRNA fold will consist of the following steps; (1) selection of a nucleotide window fragment, (2) computation of the minimum free energy fold on this fragment, (3) a check that the minimum free energy fold is not the current fold, and (4) computation of the transition rate. Steps 2 and 3 are straight-forward and can be implemented using standard dynamic programming algorithms (making sure to turn off energy contributions to multi-loops forcing hairpin only folds to be returned) while the algorithm for step 4 is discussed in later sections of the Supplementary Material. Thus, what remains to be discussed are the rules for picking window fragments.

A set of N_w window fragments used to construct folding transitions can be computed for each node in the tree. Fig D shows an example for a simple situation of an RNA containing three hairpins and no multi-loops. In Fig D(a), $N_w = 2$ and a *maximum set* of 2 window fragments are constructed for each node in the tree. For node 0, window fragment 1 corresponds to the single-stranded region 5' of the first hairpin, while window fragment 2 contains the 5' single-stranded region, hairpin 1, and single-stranded region 3' to hairpin 1. A similar construction pattern for the remaining nodes follows and is summarised in the table. The effect of increasing N_w to 4 is illustrated in Fig D(b). This results in node 0 containing 4 window fragments to check for potential re-folding, while nodes 1, 2, and 3 have 3, 2, and 1 window fragment respectively. As can be seen, with $N_w = 4$, all possible sub-fragments of the full RNA are considered. For longer RNAs, the choice of N_w will have consequences for both the computational speed and the overall number of folding reactions that will, as a result, be considered. I have used a setting of $N_w = 7$ which allows for up to 6 hairpins to be included in the "local folding" region.

Finally, Fig E shows how folding window fragments are constructed for the more general case of an RNA structure containing both hairpins and multi-loops using $N_w = 4$. The window fragment construction can be separated into two regions, region 0 containing the exterior loop and node 0 (c.f. Fig E(a)), and region 3 which contains hairpins 4 and 5 of the multi-loop helix 3 (c.f. Fig E(b)). Note, that window fragments are constructed until either a multi-loop, ribosome, or protein bound RNA structure is encountered. Thus, node 0 in Fig E(a) constructs three window fragments and stops, since the fourth fragment would contain the multi-loop (node 3 in the tree). Thus, multi-loops are treated as stable until they are melted by a ribosome.

For hairpins in a multi-loop, i.e. hairpin nodes 4 and 5 in Fig E(b), the folding windows are constructed in the same fashion as for the exterior loop (node 0), with the multi-loop represented by node 3 acting as an equivalent "node 0" for the multi-loop. Reaction propensities for the transitions can be partially summed and associated with node 0 (for reactions in region 0 - Fig E(a)) and node 3 (for reactions in region 3 - Fig E(b)).

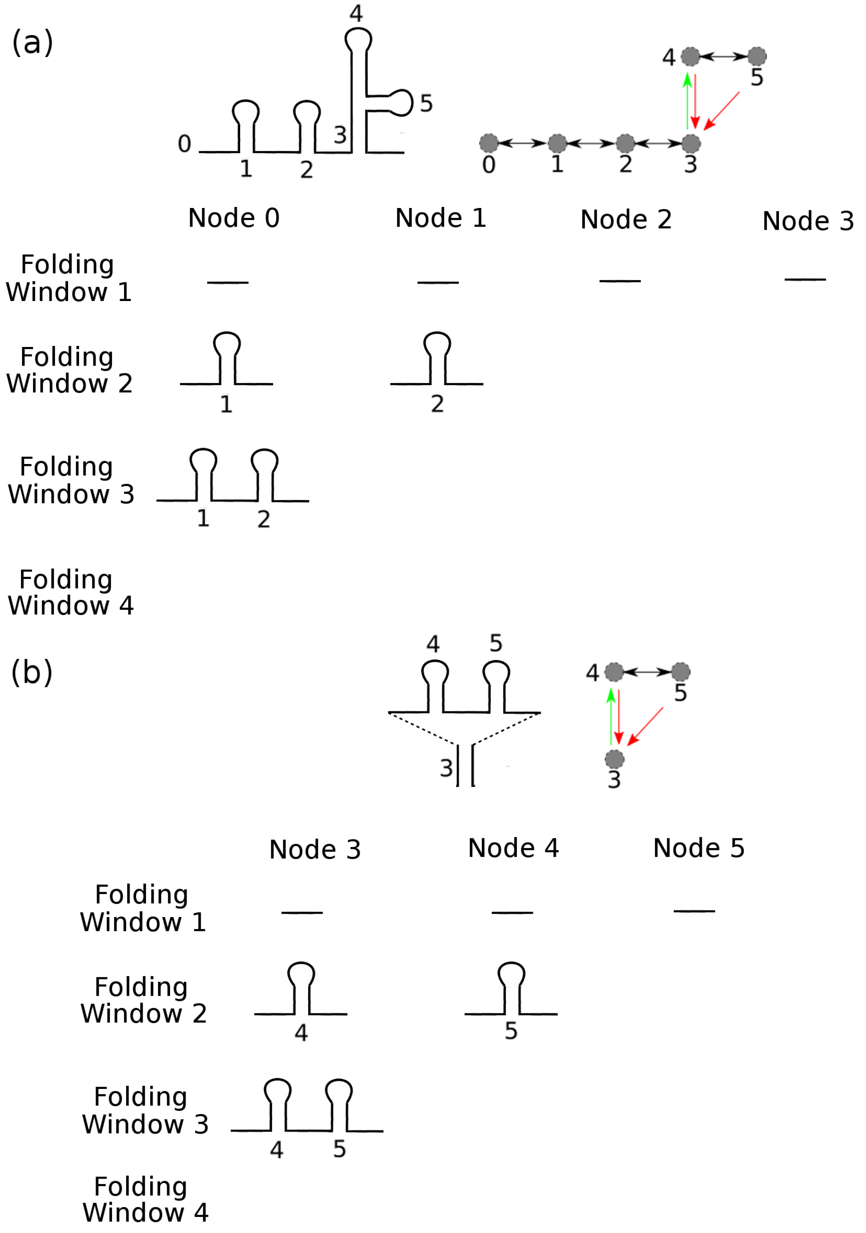

Figure E. Construction of folding windows with $N_w = 4$ for an RNA containing multi-loops. (a) Example of how folding windows are constructed for the exterior loop (region 0). Folding windows are not allowed to contain a multi-loop, ribosome, or protein bound RNA structure, hence folding window 4 is empty for node 0. (b) Example how folding windows are constructed for the multi-loop (region 3). Folding windows follow the same construction procedure as for the exterior loop.

Structural Transitions Involving Multi-Loop Formation. Due to the potential presence of the ribosome on the mRNA, the formation of multi-loops needs to be carefully considered. There are two key issues. First is that, given a ribosome on a mRNA with RNA secondary structures both 5' and 3' to the ribosome, what is the ability of the RNA to form multi-loop helices around ribosomes? Second, given a general structure of mRNA, how does one use windows to construct multi-loop helices? These questions are a bit more difficult to answer. With regards to the first question, it could be reasonably assumed that the helicase activity of ribosome movement on the mRNA prevents formation of multi-loop structures around the ribosome itself. However, this is only a conjecture and I am unaware of experimental evidence either way. So for simplicity, I have forbidden this type of RNA folding reaction. With regards to the second question, one could perform the same window fragment construction that I have employed to calculate local hairpin folding, but allow contribution of multi-loop energies when calculating the local

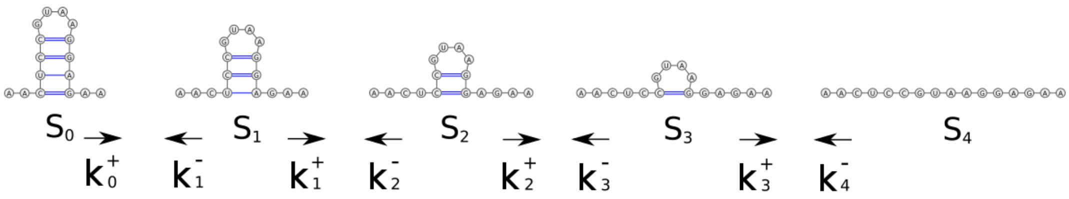

Figure F. An RNA Transition Pathway Between Two Secondary Structures The secondary structural states that the RNA transitions through are denoted by $\mathcal{S}_i$ and the kinetic rates for moving forward or backward along the path are given by k_i^+ and k_i^-, respectively.

fold. An alternative, is to give a pre-determined fixed list of allowed multi-loops based on the secondary structure input into the program. In this case, the transition structure would be constructed by adding base-pairs belonging to the multi-loops while removing any conflicting base-pairs. I have chosen the latter at this stage of development of my algorithm with plans for extending the co-translational folding algorithm to allow for formation of arbitrary multi-loops.

Estimation of the Transition Rate Between RNA Structures. Once the potential RNA folding reactions are identified using the rules stipulated in the previous sections, the folding transition rate (k_F as illustrated in Fig C) must be calculated. To do this I use techniques from the greedy RNA path search algorithm of Voss (10), and breadth-first search algorithm of Flamm (11), but use the mean-first passage time implementation discussed below.

Let $\mathcal{A}$ and $\mathcal{B}$ represent the initial and target secondary structures of the RNA respectively. To construct a transition path from $\mathcal{A} \to \mathcal{B}$ one must identify a series of additions and/or deletions of base-pairs to structure $\mathcal{A}$ which result in its transformation into structure $\mathcal{B}$. Let a specific path from $\mathcal{A} \to \mathcal{B}$ consisting of l steps be given by a series of base-pair additions/deletions

$$\mathcal{P}_l(\mathcal{A},\mathcal{B}) = (i,j),(k,l),(m,n)\ldots,$$

where (i,j) are the specific sequence of base-pair additions/deletions that form a path through the energy landscape from $\mathcal{A} \to \mathcal{B}$. In alternative terms, the path can also be described as a series of secondary structures $\mathcal{S}_i$

$$\mathcal{P}_l(\mathcal{A},\mathcal{B}) = \mathcal{S}_0,\mathcal{S}_1,\ldots\mathcal{S}_l,$$

each with energy $E(\mathcal{S}_i)$, which transition the structure $\mathcal{S}_0 = \mathcal{A}$ into $\mathcal{S}_l = \mathcal{B}$ in l steps. An additional requirement on the path $\mathcal{P}_l$ is that the sequence of base-pair additions/deletions (i,j) must not result in a base-pairing arrangement which is forbidden, such as a single nucleotide i being simultaneously paired with both nucleotides j and k. The goal of near-optimal RNA path finding algorithms is to identify a single transition path using a set of base-pair additions $(i,j) \in X_a$ and base-pair deletions $(i,j) \in X_d$.

The main differences between near-optimal RNA path finding algorithms is mostly down to the size and composition of the set of base-pair additions and deletions $(i,j) \in X = X_a \cup X_d$ which are used to construct a path, and the number of combinatorial arrangements of these moves to consider. However, there is a minimal size to the set X which must always be considered. Define the minimal set of base-pairs to delete, X_d^{min}, as the base-pairs which are contained in $\mathcal{A}$ but not in $\mathcal{B}$. Similarly, we can define the minimal set of base-pairs to add, X_a^{min}, as the base-pairs which are contained in $\mathcal{B}$ but not in $\mathcal{A}$. It is obvious that, by deleting the base-pairs X_d^{min} and adding the base-pairs X_a^{min} to structure $\mathcal{A}$, we will obtain structure $\mathcal{B}$. Indeed, any path $\mathcal{P}_l(\mathcal{A},\mathcal{B})$ which describes a transition $\mathcal{A} \to \mathcal{B}$ must include, at a minimum, addition of the set of base-pairs X_a^{min} and deletion of the set X_d^{min} as part of its sequence of moves. Direct methods only consider transition paths that are constructed using these minimal base-pair sets, while indirect methods use base-pair additions and deletions sets of which X_d^{min} and X_a^{min} are subsets.

Energy barrier and optimal transition path. The majority of RNA path finding algorithms define an optimal path as the transition path which encounters the lowest energy peak (or saddle) during the walk through the energy landscape from $\mathcal{A} \to \mathcal{B}$. Thus, the energy barrier for a path $\mathcal{P}_l(\mathcal{A},\mathcal{B})$ is typically defined as

$$E_b(\mathcal{P}_l) = Max\{E(\mathcal{S}_i)|i \in [0,l]\} - E(\mathcal{A}) \qquad [1]$$

Note that for the barrier for a path given by Eq. 1 to make sense, it must be calculated with reference to another secondary structure. As path finding algorithms are looking for a pathway from $\mathcal{A} \to \mathcal{B}$ and the energy barrier that is encountered, the natural reference structure is the starting state $\mathcal{A}$. An optimal path can thus be defined in terms of the energy barrier in Eq. 1 as the path $\mathcal{P}^*$ for which $E_b(\mathcal{P}^*)$ is minimal, i.e.

$$\mathcal{P}^* = Min\{E_b(\mathcal{P})|\mathcal{P} \in S_p\} \qquad [2]$$

where S_p is the set of all valid path transitions from $\mathcal{A} \to \mathcal{B}$ that can be constructed using the move set $X = X_a \cup X_d$.

(a) Greedypath $(\mathcal{A}, \mathcal{B})$

maxE = $E(\mathcal{A})$

Set $\mathcal{S} = \mathcal{A}$

while $\mathcal{S} \neq \mathcal{B}$

  Compute base-pairs to delete $X_d = \{(i,j) | (i,j) \in \mathcal{S}, (i,j) \notin \mathcal{B}\}$

  Compute base-pairs to add $X_a = \{(i,j) | (i,j) \in \mathcal{B}, (i,j) \notin \mathcal{S}\}$

  Compute neighbours $\mathcal{N}$ of $\mathcal{S}$ using $(i,j) \in X_a \cup X_d$

  Set $\mathcal{S}$ to neighbour $\mathcal{N}$ with lowest energy

  If $E(\mathcal{S}) >$maxE

    maxE$= E(\mathcal{S})$

return maxE

(b) Findpath $(\mathcal{A}, \mathcal{B})$

Set $\mathcal{S} = \mathcal{A}$

while $\mathcal{S} \neq \mathcal{B}$

  for $n = 0, n < k, n$++

    Compute base-pairs to delete $X_d = \{(i,j) | (i,j) \in \mathcal{S}_n, (i,j) \notin \mathcal{B}\}$

    Compute base-pairs to add $X_a = \{(i,j) | (i,j) \in \mathcal{B}, (i,j) \notin \mathcal{S}_n\}$

    Compute neighbours $\mathcal{N}$ of $\mathcal{S}_n$ using $(i,j) \in X_a \cup X_d$

    Compute $E_b(\mathcal{P}(\mathcal{A}, \mathcal{N}))$ for each neighbour

  Set $\{\mathcal{S}_n | n \in [0, k-1]\}$ to the k neighbours $\mathcal{N}$ with lowest E_b

  Update the corresponding paths $\mathcal{P}(\mathcal{A}, \mathcal{S}_n)$

  barrier = min $\{E_b(\mathcal{P}(\mathcal{A}, \mathcal{S}_n)) | n \in [0, k-1]\}$

return barrier

(c) Findpath-mfp $(\mathcal{A}, \mathcal{B})$

Set $\mathcal{S} = \mathcal{A}$

while $\mathcal{S} \neq \mathcal{B}$

  for $n = 0, n < k, n$++

    Compute base-pairs to delete $X_d = \{(i,j) | (i,j) \in \mathcal{S}_n, (i,j) \notin \mathcal{B}\}$

    Compute base-pairs to add $X_a = \{(i,j) | (i,j) \in \mathcal{B}, (i,j) \notin \mathcal{S}_n\}$

    Compute neighbours $\mathcal{N}$ of $\mathcal{S}_n$ using $(i,j) \in X_a \cup X_d$

    Compute $T_m(\mathcal{P}(\mathcal{A}, \mathcal{N}))$ for each neighbour

  Set $\{\mathcal{S}_n | n \in [0, k-1]\}$ to the k neighbours $\mathcal{N}$ with lowest T_m

  Update the corresponding paths $\mathcal{P}(\mathcal{A}, \mathcal{S}_n)$

  t = min $\{T_m(\mathcal{P}(\mathcal{A}, \mathcal{S}_n)) | n \in [0, k-1]\}$

return t

Figure G. Pseudo-code for various RNA path finding algorithms (a) *Greedypath.* Pseudo-code for the prediction of the optimal RNA transition path using the greedy method of Voss (10). (b) *Findpath.* Pseudo-code for the prediction of the optimal RNA transition path using the breadth-first search method of Flamm (11). (c) *Findpath-mfp.* Pseudo-code for the prediction of the optimal RNA transition path using the breadth-first search method of Flamm (11), but with paths selected according to those having the lowest mean first passage times.

The greedy path finding algorithm. Suppose that the minimal set of base-pair additions and deletions consist of n base-pair moves. The greedy Voss algorithm is a direct method as it attempts to construct an optimal path $\mathcal{P}_n(\mathcal{A}, \mathcal{B})$ by using the minimal base-pair move set. This method is similar to the Morgan-Higgs direct method (12), but tests addition and removal of all base-pairs in the set $X = X_a \cup X_d$, not just those with minimal clash (see Dotu for further details (13)). As the potential number of possible direct paths of length n is bounded from above by $n!$, and thus for large n, it is hardly feasible to test all possible direct pathways. The Voss algorithm attempts to identify a near-optimal path by selecting the secondary structure at each step i which has minimal energy. In this way, the algorithm attempts to put an upper bound on the barrier E_b by always choosing the next structure in the path sequence $\mathcal{S}_i$ to have smallest energy. In the first step, the algorithm constructs up to n secondary structures using the starting structure and the set of base-pair moves which do not result in base-pair clashes. From this set of up to n structures, the structure which has lowest energy is selected as the next state in the path. In subsequent steps, new structures are constructed from the previous structures in the path using the remaining moves. The total computational cost of this method scales as $O(n^2)$ during the construction of the transition pathway. Pseudo-code for the algorithm is given in Fig G(a). Although intuitive, this method does not ensure the path with lowest energy barrier (with E_b defined using Eq. 1 and optimal path by Eq. 2) will be identified. This is because there may be some paths through the energy landscape that, by selecting a structure with a larger increase in energy early on in the path, may result in an overall pathway which has lower energy barrier.

The breadth-first search path finding algorithm. The observation that some paths which start down a higher energy pathway may result in a lower energy barrier for the path overall led to the development of a breadth-first search approach to the Voss algorithm (10). This is implemented by Findpath in the Vienna RNA package (14). Essentially, at each step of the path construction, up to k lowest energy paths are retained. Thus, a breadth-first-search of the energy landscape is performed and the total computational cost scales as roughly $O(kn^2)$. Pseudo-code for the algorithm is shown in Fig G(b). Once the barrier has been calculated using the Findpath algorithm, programs such as Kinwalker (8), which predicts coarse-grained co-transcriptional folding of RNA, rely on the following approximation for the transition time in terms of the predicted energy barrier E_b for the path

$$\begin{aligned} t &= 10^x \\ x &= \frac{8E_b - 77}{11}. \end{aligned} \quad [3]$$

Identifying optimal paths using mean first passage time. Instead of using the barrier definition of Eq. 1 and an optimal pathway given by Eq. 2, I propose using an alternative method for identification of an optimal pathway using the mean first passage time. As I will show, this will enable an estimation of the mean kinetic transition rate k_F (c.f. Fig C), which will be needed in the stochastic co-translational folding model. Consider a single transition path from $\mathcal{A} \to \mathcal{B}$, i.e. $\mathcal{P}_l(\mathcal{A}, \mathcal{B})$ which transitions through a set of secondary structures $\mathcal{S}_i$. Let ΔG_i denote the change in free energy that occurs during the transition from secondary structure $i-1$ to i,

$$\Delta G_i = E(\mathcal{S}_i) - E(\mathcal{S}_{i-1}).$$

If this pathway was the only possible way for the RNA to transition from $\mathcal{A} \to \mathcal{B}$, then the pathway is simply a one-dimensional chain of states, and the transition kinetics can be computed using Markov chain analysis (15). In such a situation, the mean first passage time t_m for the transition $\mathcal{A} \to \mathcal{B}$ along the path $\mathcal{P}_l(\mathcal{A}, \mathcal{B})$ can be computed recursively using the formulas:

$$t(l-1) = \frac{1}{k^+_{l-1}} \quad [4]$$

$$t(i) = \frac{1}{k^+_i} + \frac{k^-_{i+1}}{k^+_i} t(i+1) \quad 0 \le i < l-1 \quad [5]$$

$$t_m = \sum_{i=0}^{l-1} t(i) \quad [6]$$

where k^+_i and k^-_i are the kinetic rates for transitioning from state i to states $i+1$ and $i-1$, respectively, and the $t(i)$ are the average time spent in state i in a mean first passage from state $0 = \mathcal{A} \to \mathcal{B} = l$. The kinetic rates can be approximated using the approximations used by Kinfold and KFOLD (6, 7) for single base-pair folding kinetics, i.e.

$$k^+_{i-1} = Ae^{-\beta \Delta G_i/2} \quad [7]$$

$$k^-_i = Ae^{+\beta \Delta G_i/2}, \quad [8]$$

where A is the attempt frequency and $\beta = 1/k_b T$. Equations 7 and 8 are the Kawasaki rules for calculating forward and backward rates (16) and have been used to compute RNA folding kinetics at single base-paired resolution with good accuracy (6, 7). With a small amount of algebraic manipulation of Eqs. 4-6, one can see that the Kawasaki rules will scale the overall mean first passage time via A as $t_m \propto 1/A$. Finally, since

$$t(i) = \frac{1}{k^+_i} + t(i+1)e^{\beta \Delta G_{i+1}},$$

the Kawasaki rules (or any other method for deducing forward and backwards rates from the total free energy difference) will effect t_m via the estimation of the forward rates between states k^+.

Let $t_m = T_m(\mathcal{P})$ denote the mean first passage time for the path computed using Eqs. 7-9. Then an optimal path can now be defined as the path $\mathcal{P}^*$ which satisfies

$$\mathcal{P}^* = Min\{T_m(\mathcal{P}) | \mathcal{P} \in S_p\}. \quad [9]$$

It should be noted that since there will be multiple possible pathways from $\mathcal{A} \to \mathcal{B}$, the true mean first passage time will be an average over the mean first passage times for all possible pathways, weighted by their probabilities of occurrence. Thus, the mean free passage time reported here is an approximation, and can be thought of as rough lower bound on the true mean first passage time. Senter and Clote (17) have utilised a more computationally expensive fast Fourier transform based method for computing the exact mean first passage time between two structures, taking into account multiple transition pathways. However, since a single "best" pathway is what is required here, the approximate method is a sufficient tool for comparing the pathways between each other and estimating the *apparent* kinetic barrier $E^\dagger_b(\mathcal{P})$ that is encountered, i.e.

$$E^\dagger_b(\mathcal{P}) = \frac{1}{\beta} \ln\left(\frac{T_m(\mathcal{P})}{A}\right),$$

where A is the attempt frequency. Implementation of the mean first passage time, within a breadth-first search algorithm, can be described by the pseudo-code in Fig G(c). Once the optimal pathway $\mathcal{P}^*$ and its mean first passage time have been calculated using the breadth-first search algorithm in Fig G(c), the mean kinetic transition rate k_F between states $\mathcal{A} \rightarrow \mathcal{B}$ can be estimated as

$$k_F = \frac{1}{T_m(\mathcal{P}^*)}. \quad [10]$$

Comparison with predictions from KFOLD. The breadth-first path searching algorithm described in Fig G(c) constructs a single direct pathway from which the mean first passage time is estimated using equations 4-8. However, the true mean first passage time requires calculation of a Boltzmann weighted sample of all possible folding pathways between the two states of interest. As such, a natural question arises as to the accuracy the mean first passage time calculated from a single direct path. To probe this question, I have calculated the mean first passage times of the wild-type and mutant 11 MS2 coat hairpins (c.f. Fig 3A in main text) using the KFOLD program. These were chosen as they where the least thermodynamically stable hairpins allowing KFOLD to predict the melting time in a reasonable amount of computational time. The KFOLD program allows all possible single base-pair additions and deletions which alter the hairpin structure to be used to construct a transition pathway between the folded and single-stranded states. Thus, KFOLD is able to probe indirect paths and more complicated paths which pass through multiple minima. The melting of the coat hairpin structure was simulated in KFOLD using Kawasaki rules (Eqs. 7 and 8) using an attempt frequency $A = 3.0 \times 10^7$ s^{-1}. A total of 5000 melting pathways were constructed and the total simulation time logged. S3 Table, which summarises the results, demonstrates that the breadth-first search algorithm is able to provide reasonable predictions of the mean first passage time. There is a noted discrepancy with mutant 11 at 37° C. There are two possibilities for this deviation. The first is that an indirect pathway involving base-pairs which are not present in the structure dominates at 37°. This would be picked up by the KFOLD algorithm, but not the breadth-first search since it is a direct path algorithm. The second, and more likely, possibility is that the sampling was insufficient and that paths with long unfolding times where under-sampled. Since KFOLD may take up to an hour of computer to sample a path with time $>$ 10s, an upper limit of 15s was set on the maximum fold time. It may be that there exist substantial number of unfolding paths which are above this threshold and the results have been skewed as a result.

Interaction of ribosomes and proteins with mRNA

In this section, I discuss a model for 30S:PIC binding to mRNA and the binding of MS2 coat protein to the RNA. For the construction of the 30S:PIC binding model, I use insights from the bacteriophage MS2 genomic RNA and the regulation of ribosome initiation on its coat gene, where 30S:PIC binding depends on the local secondary structure of the translation initiation region (TIR) and the strength of the coat operator hairpin. Although insights from bacteriophage MS2 are used to construct the model, I believe that it should be suitable for general use. Finally, I discuss the model used for MS2 coat protein binding to RNA.

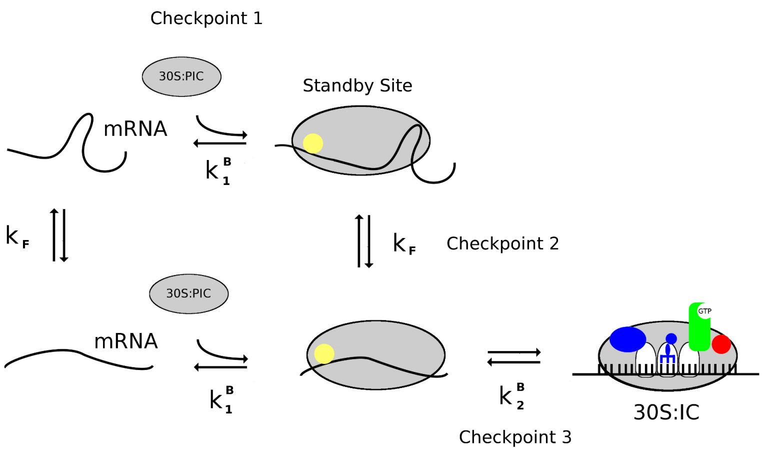

Figure H. Kinetic model of 30S:PIC binding to mRNA to form the 30S Initiation Complex. The initial binding of 30S:PIC to mRNA with rate K_1^B proceeds via recognition of ribosomal protein S1 (yellow dot) followed by recognition of the Shine-Dalgarno sequence and start codon. The model includes two pathways to formation of the 30S:IC: (1) the standby pathway, where the 30S:PIC first binds to a weakly structured area of the mRNA and waits until RNA unfolding presents the start codon, and (2) a pathway in which the 30S:PIC binds to unstructured RNA.

Model of 30S:PIC binding to mRNA. During initiation of the ribosomal translational machinery on an mRNA, the 30S:PIC binds to a TIR to form the 30S initiation complex (30S:IC) and then undergoes subsequent maturation to become a 70S elongation complex (70S:EC) after completing a series of kinetic checkpoints that ensure proper identification of, and initiation on, the correct start codon. There is some evidence that the initial binding of the 30S:PIC subunit to mRNA takes place via the ribosomal S1 protein, which has strong preference for binding non-sequence specifically to single-stranded RNA (18). Although

S1 tends to have a preference for binding large stretches of single-stranded nucleotides ($\geq$ 20 nt) with an association constant of roughly $K_a = 3 \times 10^6 M^{-1}$ (18), it has been shown to also be capable of binding to weakly structured areas containing a few hairpins (19, 20). Moreover in bacteria, access to the TIR tends to be regulated by mRNA secondary structure, which can sequester the start codon in base-paired regions of the mRNA, preventing the recognition of the start codon by the 30S:PIC. Such structured areas in TIRs are known to exist in the RNA bacteriophages MS2 and Qβ (21) and van Duin and colleagues have shown that translation initiation would be extremely poor/slow if the ribosome was forced to wait until the RNA structure around the coat gene in MS2 unfolded (20). Thus, it has been postulated that standby sites, *i.e.* areas of weak secondary structure that the 30S:PIC is capable of binding to, allow the 30S:PIC to wait on the mRNA until unfolding of the TIR occurs (20).

My model of 30S:PIC binding incorporates the standby state into the 30S:PIC binding model along with a standard binding pathway to unstructured mRNA as shown in Fig H. This can be described by a four state model for both structured and un-structured mRNAs as depicted in Fig I(a). The binding pathway is equivalent to that of van Duin's (20), with an additional recognition step with kinetic rate k_2^B that corresponds to the third kinetic checkpoint in (22). Thus, I have modelled the binding as a two-step process with the initial step of 30S:PIC binding to the mRNA mediated by ribosomal protein S1 (with kinetic rate k_1^B), followed by the subsequent step of codon recognition and binding to any Shine-Dalgarno region on the mRNA (with kinetic rate k_2^B).

Dependence of Initiation Efficiency on the Kinetic Checkpoints 1-3. Here I describe how the ribosome initiation efficiency on an mRNA is taken into account in the 30S:PIC binding model (Fig H and Fig I) based on its explicit nucleotide sequence and the kinetic rates k_F, k_1^B and k_2^B. In total, there are 5 kinetic checkpoints (22, 23) in which the sequence and structure of the mRNA can alter the kinetics of initiation, thereby affecting the translational initiation efficiency on a given mRNA sequence. In this subsection, I focus on the three kinetic checkpoints 1-3, illustrated in Fig H, which take into account the local RNA secondary structure of the TIR. Previously, Salis (24) reported on an estimate of the translational initiation rate based on the stability of mRNA structure and the energetics of interaction with an SD site in the mRNA. The Salis model estimates the total initiation rate from $r \propto e^{\beta \Delta G_{tot}}$, where

$$\Delta G_{tot} = \Delta G_{mRNA:rRNA} + \Delta G_{start} + \Delta G_{spacing} - \Delta G_{standby} - \Delta G_{mRNA}$$

is a sum of the free energy changes which takes place during TIR melting and interaction of the ribosome with the mRNA. The first three terms describe the interaction of the 30S subunit with the mRNA, while the last two terms describe the energetic cost to melt the mRNA structure which hides the standby site ($\Delta G_{standby}$) or the TIR region (ΔG_{mrna}). In my 30S:PIC binding model, the initiation rate will depend on the time that it takes for the 30S:PIC to pass through kinetic checkpoints 1-3 which depend on the mRNA sequence and its structure. Specifically these are: (1) the initial *non-sequence specific* binding of the 30S:PIC to mRNA - k_1^B, (2) mRNA unfolding rate - k_{-F}, and (3) sequence specific recognition of the SD sequence and start codon by 16S rRNA - k_2^B. These three rates determine the apparent rate for 30S:PIC binding to the TIR, k_{on}. Values for k_{on} and k_{off} have been experimentally measured for small RNA fragments by Studer and Joeseph (19). In these experiments, the off rates ranged from $0.0001 s^{-1}$ to $4.0 s^{-1}$ while the on rates ranged from 1 to $250 \mu M^{-1} s^{-1}$, depending on the presence of an SD sequence (or partial SD sequence), melting temperature of the mRNA, and existence of a standby site. The goal here is to choose values of k_1^B and k_2^B so that overall off and on rates for the ribosome are within ranges estimated by Studer and Joeseph (19).

Thus, the determination of the apparent on and off rates of 30S:PIC binding/un-binding from the mRNA (k_{off}) in my model depends on the interaction of *both* the S1 protein as well as the interaction of the start codon and any Shine-Dalgarno sequence with the 30S subunit. An energy profile of this interaction ($\Delta G_{30S:mRNA}$) as the 30S:PIC subunit moves through states 1-4 is shown in Fig I(b). In stages 2 and 3 (corresponding to checkpoint 1), $\Delta G_{30S:mRNA}$ is equal to the interaction of S1 with mRNA, while in stage 4,

$$\Delta G_4 = \Delta G_{mRNA:rRNA} + \Delta G_{start} + \Delta G_{spacing} \, .$$

which is the total energy of interaction of the 30S with the SD sequence, start codon along with any penalty from incorrect spacing between the SD sequence and start codon. Studer and Joseph estimated off rates of the 30S:PIC from un-structured mRNA lacking any Shine-Dalgarno sequence to be on the order of $k_{off} = 1.0$ to $4.0 s^{-1}$, while off rates for mRNAs containing the full Shine-Dalgarno sequence where roughly $k_{off} = 0.0001$ to $0.006 s^{-1}$. Here I assume that for mRNA sequences lacking a Shine-Dalgarno sequence, the *apparent* 30S:PIC off-rate is essentially equivalent to k_{-1}^B, and that this binding is mediated by the ribosomal protein S1. This protein has been shown to bind to single-stranded regions of mRNA (18) and has also been shown to play a role in standby site binding (20). Draper and von Hipple estimate that the intrinsic binding affinity of S1 for single stranded RNA is roughly $K_a = 3 \times 10^6$ M^{-1}. Thus, it can be expected that the binding energy of S1 for ssRNA is approximately $\Delta G_{S1} = -9.19$ kcal/mol, which is consistent with estimates for average binding energy of ribosome 30S subunit for mRNA that De Smit and van Duin (25), along with Gualerzi *et al.* (26) have estimated based on experiments. Hence, I assume that

$$\frac{k_1^B}{k_{-1}^B} = 3.0 \times 10^6 = e^{-\beta \Delta G_{S1}} \, .$$

I find that $k_1^B = 30 \mu M^{-1} s^{-1}$ with $k_{-1}^B = 10 s^{-1}$ provides a reasonable ratio that recapitulates the experimental measured ratios from van Duin of coat protein in MS2 (25) (see discussion in main text) along with the expected free ribosome levels over the whole cell (i.e. 15%) compared to the number of mRNAs for various cell growth rates.

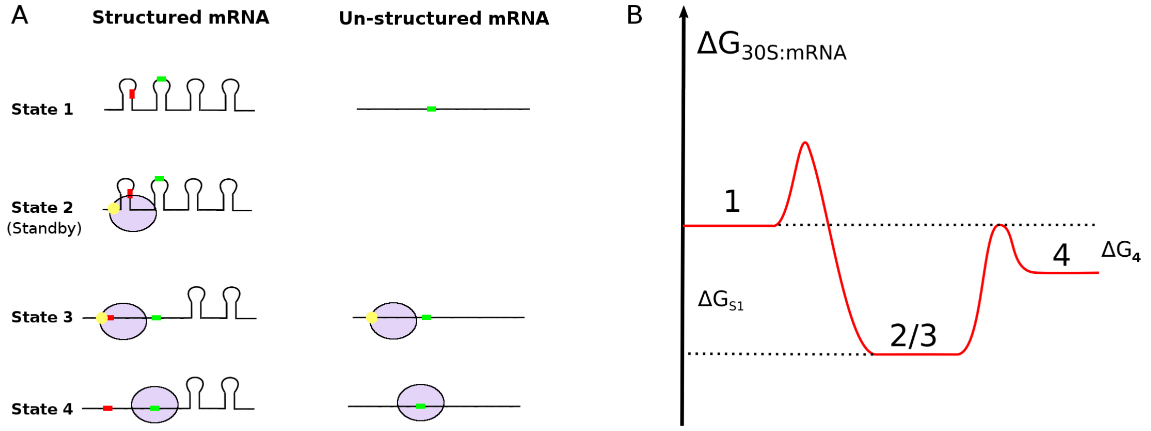

Figure I. Energy profile of 30S:PIC binding to mRNA. (A) The binding of the 30S:PIC subunit to mRNA can take place via two pathways, one which depends on structured mRNA where 30S:PIC binds via a standby site (State 2) and a second, where mRNA is mostly unstructured and the 30S:PIC skips a standby state. (B) Model of the energy profile of 30S:PIC interaction with the mRNA. In states 2 and 3, the 30S subunit is modelled as interacting with mRNA predominately via ribosome protein S1 (yellow dot), where in state 4, the interaction is directly with the start codon (green bar = start,red bar = stop) and any Shine-Dalgarno sequence present in the mRNA.

The kinetic rates of folding/unfolding of the TIR, k_F and k_{-F}, which govern checkpoint 2 in Fig H are computed from

$$\frac{k_F}{k_{-F}} = e^{-\beta \Delta G_F} \quad [11]$$

$$k_F = A e^{-\beta \Delta G^{\dagger}} \quad [12]$$

where the factor $A = 3 \times 10^7$ s^{-1} is the attempt frequency (20) and $\Delta G^{\dagger}$ is the energetic barrier to folding. The value ΔG_F is the total change in free energy due to mRNA unfolding of the TIR and is approximately equivalent to the value $\Delta G_{mRNA} + \Delta G_{standby}$ that is calculated by Salis. A value for $\Delta G^{\dagger}$ can be calculated using the mean-first passage time implementation of Findpath (see above), which identifies an optimal kinetic pathway between two RNA structures with minimum mean first passage time. Energetic parameters used for the RNA base stacking calculations are the Turner 99 nearest neighbour parameters (27), and kinetic rates between states along the unfolding pathway are calculated from Eqs. 7 and 8.

The kinetic rates for sequence specific recognition of the Shine-Dalgarno sequence and start codon (checkpoint 3 in Fig H) are estimated using the relation

$$\frac{k_2^B}{k_{-2}^B} = e^{-\beta(\Delta G_4 - \Delta G_{S1})} = e^{-\beta \Delta x} \quad [13]$$

where in stage 4,

$$\Delta G_4 = \Delta G_{mRNA:rRNA} + \Delta G_{start} + \Delta G_{spacing}$$

is the energy of interaction of the fMet codon with mRNA (ΔG_{start}), plus interaction of the 16S rRNA with the SD sequence in the mRNA ($\Delta G_{mRNA:rRNA}$). As discussed above, ΔG_{S1} is the non-sequence specific binding energy of the S1 protein to mRNA. Thus, this assumes that in the process of binding to the start codon at checkpoint 3, ribosomal protein S1 must dis-engage from the mRNA. Note, without this assumption, the total binding energy of S1 combined with the SD interaction resulted in off rates of the 30S subunit which were extremely slow, 100 times smaller than what was measured by Studer and Joesph (19). Thus, in this model the total 30S binding energy to the mRNA ranges from -9.19 to -13.6 kcal/mol, depending on the SD sequence. The energy of interaction of the fMet-tRNA anti-codon with the start codon ΔG_{start}, along with the energetic cost of non-optimal spacing between the SD site and the start codon, $\Delta G_{spacing}$ are calculated as follows. The interaction with the start codon ΔG_{start} can be obtained from Turner's nearest neighbour parameters and is roughly -1.2 kcal/mol for AUG start codons. For $\Delta G_{spacing}$, I use the formula from Salis et al. (24),

$$\Delta G_{spacing} = \begin{cases} 0.048(s-5)^2 + 0.24(s-5) & s \geq 5 \\ 12.2\left(1 + e^{2.5(s-3)}\right)^{-3} & s < 5 \end{cases}, \quad [14]$$

where s is the number of nucleotides between the 3' end of the SD sequence and the first nucleotide in the start codon. For an ideally spaced SD sequence and canonical AUG start codon, $s = 5$ and $\Delta G_{spacing} = 0$.

It is difficult to estimate a value for the rate of transfer of the 30S to the start codon, k_2^B, as there is limited experimental information in the literature on the individual kinetic steps that take place during the initial ribosome binding to the mRNA. As will be shown in the next section, a value for k_2^B can be estimated by fitting to the range of apparent off rates experimentally measured by Studer and Joeseph (19).

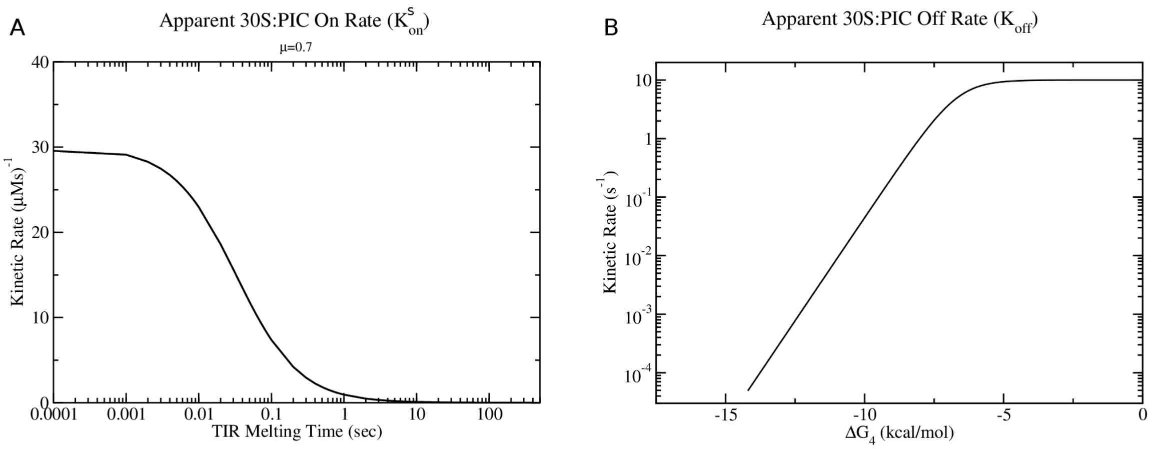

Figure J. Apparent kinetic rates of 30S:PIC binding to mRNA. (A) Plot of Equation 16 for varying TIR melting times. The free concentration of 30S:PIC was set to 0.69 μM, following the predicted estimates from my Ribosome model (1) at a growth rate of $\mu = 0.7$ doublings per hour. (B) Plot of Equation 17 for varying strength of the interaction between the Shine-Dalgarno sequence with rRNA (ΔG_4) using values $k^B_{-1} = 10$ s^{-1} and $\Delta G_{S1} = -9.19$ kcal/mol with $\Delta x = \Delta G_4 - \Delta G_{S1}$.

Formula for Apparent 30S Binding Rates to mRNA. Although the binding of 30S:PIC subunits onto the mRNA in this model follows the kinetic steps depicted in Fig H, one can derive simplified equations for the apparent on and off rates k_{on} and k_{off} using mean first passage times and Markov chain analysis. For an mRNA lacking a standby site, the predominant binding pathway is that of the lower path in Fig H, while for mRNAs having a standby site, it is the upper pathway. Since most secondary structure (even weak secondary structure) will take at least on the order of several 100s of microseconds to unfold, it is reasonable to assume that $k^B_2 >> k_{-F}$, and that the rate limiting step in 30S:PIC binding to mRNA is the rate of mRNA unfolding k_{-F}. Thus, for mRNAs lacking a standby site, the mean first passage time of 30S:PIC binding to mRNA (τ_{on}) can be calculated as

$$\begin{aligned} \tau_{on} &= \frac{1}{r_f} + \frac{k_F}{k_{-F}}\frac{1}{r_f} + \frac{1}{k_{-F}} \\ &= \frac{r_f + k_{-F} + k_F}{r_f k_{-F}} \,. \end{aligned}$$

Similarly, for mRNAs with a standby site, the mean first passage time of 30S:PIC binding (τ^s_{on}) can be computed as

$$\begin{aligned} \tau^s_{on} &= \frac{1}{r_f} + \frac{k^B_{-1}}{r_f}\frac{1}{k_{-F}} + \frac{1}{k_{-F}} \\ &= \frac{k_{-F} + k^B_{-1} + r_f}{r_f k_{-F}} \,. \end{aligned}$$

Here, the value of $r_f = k^B_1[\text{30S}]$ is the rate of S1 binding to mRNA times the concentration of free 30S:PIC subunits. Using $k_{on}[\text{30S}] \approx 1/\tau_{on}$ along with the time for TIR unfolding, $\tau_u = 1/k_{-F}$ we can estimate the apparent binding rates as

$$k_{on} = \frac{k^B_1}{1 + e^{-\beta \Delta G_F} + \tau_u r_f} \quad [15]$$

$$k^s_{on} = \frac{k^B_1}{1 + \tau_u (r_f + k^B_{-1})} \,. \quad [16]$$

Similar to above, we can derive the mean first passage time for 30S:PIC unbinding as

$$\begin{aligned} \tau_{off} &= \frac{1}{k^B_{-1}} + \frac{k^B_2}{k^B_{-2}}\frac{1}{k^B_{-1}} + \frac{1}{k^B_{-2}} \\ &= \frac{1}{k^B_{-1}}\left(1 + \frac{k^B_2}{k^B_{-2}}\right) + \frac{1}{k^B_{-2}} \,. \end{aligned}$$

For a range of ΔG_4 values, i.e. $[-13.6, 0]$ kcal/mol, values of k_{off} should range between $0.0001 < k_{off} < 4$ to be consistent with measurements of Studer and Joeseph (19). Setting $k^B_{-2} = 0.5 \times 10^6 e^{\beta \Delta G_4}$ gives an off rate of $k_{off} \approx 0.0001$ s^{-1} when $\Delta G = -13.6$ kcal/mol. This gives the apparent k_{off} for 30S:PIC unbinding as

$$k_{off} = \frac{k^B_{-1} k^B_{-2}}{k^B_{-1} + k^B_{-2}(1 + e^{-\beta \Delta x})} \quad [17]$$

$$k^B_{-2} = 0.5 \times 10^6 e^{\beta \Delta G_4} \,. \quad [18]$$

Equations 15-18 give the apparent on and off rates for 30S:PIC binding to the mRNA both with and without a standby site present. Fig J plots the apparent on rate for different TIR melting times (Fig J(a)) along with the apparent off rate for different ΔG_4 values (Fig J(b)).

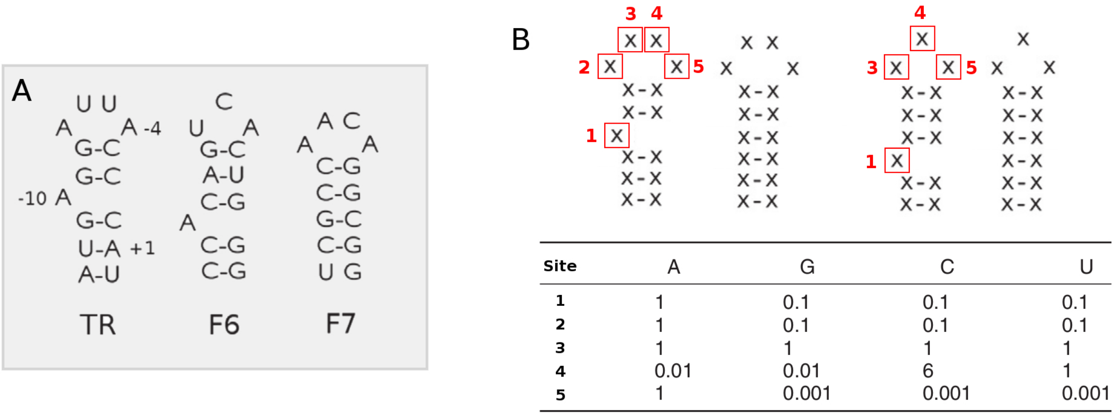

Site	A	G	C	U
1	1	0.1	0.1	0.1
2	1	0.1	0.1	0.1
3	1	1	1	1
4	0.01	0.01	6	1
5	1	0.001	0.001	0.001

Figure K. The three families of RNA hairpins which bind bacteriophage MS2 coat protein. (A) The TR, F6, and F7 hairpin variants which have been shown to bind MS2 coat protein. Sequence preferences are denoted in the figure with N = any nucleotide, Y= Pyrimidine, R = Purine. (B) Affinity matrix estimated from stop flow kinetic assays on binding of MS2 coat protein to the TR hairpin (29). The matrix can be used to estimate the change in binding affinity that results from sequence or structural changes.

Model of coat protein binding to mRNA. In bacteriophage MS2, the coat protein is able to bind to small hairpin structures in RNA that contain specific sequence and structural motifs. Fig K(a) shows the three different structural families that coat protein is known to be able to bind to. The bacteriophage MS2 coat protein has preference for RNA hairpins containing motifs in the apical loop of NNYA, where N denotes any nucleotide and Y denotes a pyrimidine. My colleagues and I have worked out a binding affinity matrix (28), shown in Fig K(b), which roughly estimates the changes in binding affinity based on the specific nucleotide sequence present in the hairpin relative to the TR hairpin. I model the kinetic rate of coat protein unbinding from a generic hairpin structure (c.f. Fig K(b)) using the formula

$$k_{off} = \frac{k_{off}^{tr}}{r_a}, \qquad [19]$$

where $\beta = 1/k_b T$ and r_a is a binding affinity ratio that is calculated using the affinity matrix in Fig K(b). To calculate the ratio r_a for a general hairpin, one takes the specific sequence present in the hairpin at each site (1 to 5) and identifies the appropriate values in the affinity matrix based on the sequence. Multiplying these values together one obtains the resulting r_a value, which is the ratio of binding affinity between the TR sequence and the hairpin. The value $k_{off}^{tr} = 1/60s^{-1}$ is the measured on/off rate of coat protein binding to TR (29). For the on rate, I use the measured on rate from stop flow binding kinetics $k_{on} = 11\mu M^{-1}s^{-1}$ (29) which is modelled as being the same for all hairpins.

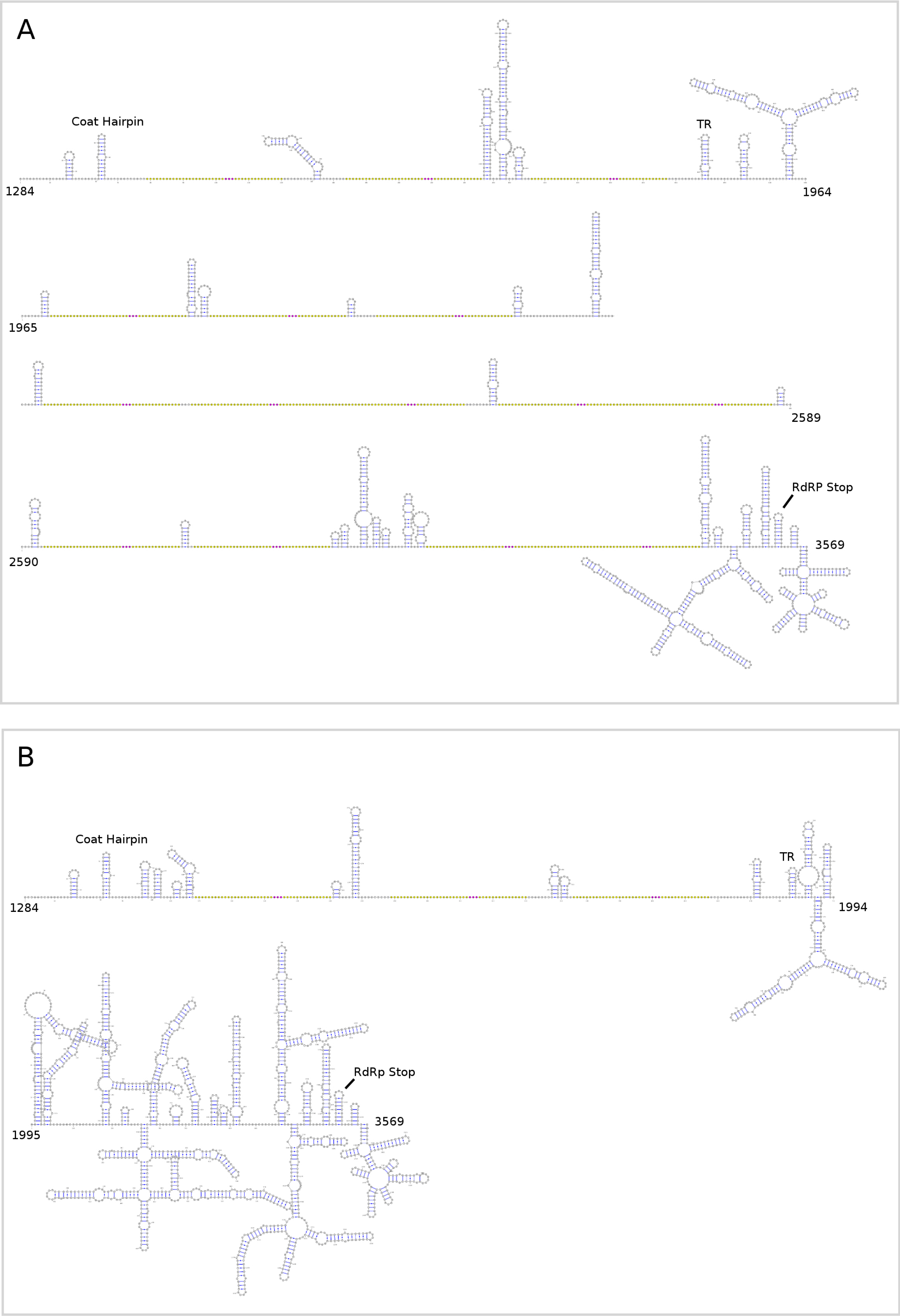

Figure L. Snapshots of MS2 mRNA secondary structure of the coat and RdRp genes during translation. (A) Secondary structure of MS2 mRNA at low coat protein concentrations when both the coat and RdRp genes are being actively translated by ribosomes. Yellow nucleotides indicate the footprint of ribosomes while purple nucleotides indicate the location of the ribosome P-site. Important secondary structures (coat hairpin, TR stem-loop, RdRp Stop Hairpin) are labelled. Numbers indicate nucleotide number in the MS2 viral RNA. (B) Secondary structure of MS2 mRNA at high coat protein concentrations when only the coat gene is being actively translated by ribosomes. The structure of the RdRp gene has re-formed long-distance interactions and has no actively translating ribosomes, while the coat gene has three ribosomes in active translation.

Table A. Numbers of various tRNAs per cell and their codon recognition. Data for the number of tRNAs at different growth rates have been adjusted to match the codon biases of the mRNAs from E. coli K12 (strain MG1655 - uniprot accession code U00096). The total tRNA at each growth rate have been normalised to overall expected total tRNA concentrations discussed in Bremer (3).

tRNA	Codon Rec.	$\mu = 0.7$	$\mu = 1.06$	$\mu = 1.50$	$\mu = 2.5$	$1/R$
Lys	AAA,AAG	3065	6131	9593	23672	0.4073
Asn	AAC,AAU	2731	5463	8548	21091	0.3629
Thr4	ACA,ACU,ACG	1475	2950	4616	11391	0.1960
Thr1+3	ACC,ACU	1573	3146	4922	12147	0.2090
Thr2	ACG	703	1406	2200	5428	0.0934
Arg4	AGA	180	361	565	1394	0.0240
Ser3	AGC,AGU	1728	3456	5408	13344	0.2296
Arg5	AGG	117	234	367	906	0.0156
Ile2	AUA	296	593	928	2289	0.0394
Ile1	AUC,AUU	3887	7775	12166	30018	0.5165
MetF	AUG	2529	5058	7914	19528	0.3360
MetM	AUG	1742	3485	5452	13454	0.2315
Gln1	CAA	1072	2145	3356	8282	0.1425
His	CAC,CAU	1578	3157	4939	12187	0.2097
Gln2	CAG	2020	4042	6324	15605	0.2685
Pro3	CCA,CCU,CCG	1605	3211	5024	12396	0.2133
Pro2	CCC,CCU	514	1029	1611	3975	0.0684
Pro1	CCG	965	1931	3022	7456	0.1283
Arg3	CGG	374	749	1173	2894	0.0498
Arg2	CGU,CGC,CGA	3254	6509	10185	25131	0.4324
Leu3	CUA,CUG	2058	4117	6442	15895	0.2735
Leu2	CUC,CUU	1546	3093	4840	11943	0.2055
Leu1	CUG	1915	3831	5994	14791	0.2545
Glu2	GAA,GAG	4013	8027	12559	30989	0.5332
Asp1	GAC,GAU	3574	7149	11186	27601	0.4749
Ala1B	GCU,GCA,GCG	4834	9669	15129	37330	0.6423
Ala2	GCC	1788	3577	5596	13809	0.2376
Gly2	GGA,GGG	834	1669	2621	6445	0.1109
Gly3	GGC,GGU	3800	7601	11892	29344	0.5049
Gly1a	GGG	485	971	1519	3748	0.0645
Val1	GUA,GUG,GUU	3061	6122	9579	23637	0.4067
Val2ab	GUC,GUU	1860	3721	5822	14367	0.2472
Tyr1+2	UAC,UAU	1976	3953	6185	15262	0.2626
Ser1	UCA,UCU,UCG	1117	2235	3497	8630	0.1485
Ser5	UCC,UCU	779	1558	2437	6015	0.1035
Ser2	UCG	407	815	1276	3150	0.0542
Cys	UGC,UGU	810	1621	2536	6259	0.1077
Trp	UGG	1064	2128	3330	8218	0.1414
Leu5	UUA,UUG	1281	2563	4011	9897	0.1703
Phe	UUC,UUU	2709	5419	8479	20923	0.3600
Leu4	UUG	636	1273	1992	4916	0.0846

Table B. Predicted and measured tRNA^{Lys} misreading frequencies. The misreading frequency by tRNA^{Lys} per 10000 reads at various near-cognate codons is given. Experimental measurements are obtained from (5) and are compared with the model at different growth rates.

Codon	Amino Acid	Misread Pos.	Exp.	Th. ($\mu = 0.7$)	Th. ($\mu = 1.0$)	Th. ($\mu = 2.5$)
UAA	Ter	1	4.1	4.2	4.4	4.2
UAG	Ter	1	14	12	12	11
AUA	Ile	2	3.5	5.6	7.7	8.0
AGA	Arg	2	36	9.0	10	11
AGG	Arg	2	31	10	17	16
AAU	Asn	3	16	0.91	0.95	0.93
AAC	Asn	3	3.8	0.91	0.95	0.93
AUG	Met	2	3.1	1.5	1.3	1.4
CAA	Gln	1	3.1	1.9	2.3	2.4
CAG	Gln	1	3.1	1.2	1.3	1.3

Table C. Mean first passage times for MS2 coat hairpin unfolding. The mean first passage times (τ) are calculated using two different methods (1) a calculation using 5000 simulations of KFOLD, column KFOLD in table and (2) a calculation using the breadth-first search algorithm in Fig G(c) (column BFS in table). Temperatures are in degrees Celsius while mean first passage times are in seconds. The discrepancy for Mutant 11 at 37° may be due to paths with longer times being under-represent in the sampling, or an indirect pathway which dominates.

Coat Hairpin	Temp.	KFOLD	BFS
Wild-type	37	2.11	3.37
Mutant 11	37	4.31	13.53
Wild-type	42	0.18	0.30
Mutant 11	42	0.60	0.89

References

1. Dykeman EC (2020) A stochastic model for simulating ribosome kinetics *in vivo*. *PLOS Comp Biol* 16(2):e1007618.
2. Dong H, Nilsson L, Kurland CG (1996) Co-variation of tRNA Abundance and Codon Usage in *Escherichia coli* at Different Growth Rates. *J Mol Biol* 260:649-663.
3. Bremer H, Dennis PP (2008) Modulation of Chemical Composition and Other Parameters of the Cell at Different Exponential Growth Rates. *Ecosal Plus 2013*; doi: 10.1128/ecosal.5.2.3
4. Dai X, Zhu M, Warren M, Balakrishnan R, Patsalo V, Okano H, Williamson JR, Fredrick K, Wang YP, and Hwa T (2016) Reduction in translationg ribosomes enables *Escherichia coli* to maintain elongation rates during slow growth. *Nat Microbiology* 2:16231.
5. Kramer EB and Farabaugh PJ (2007) The frequency of translational misreading errors in *E. coli* is largely determined by tRNA competition. *RNA* 13(1):87-96.
6. Flamm C, Fontana W, Hofacker IL, and Schuster P (2000) RNA folding at elementary step resolution. *RNA* 6(3):325-338.
7. Dykeman EC (2015) An implementation of the Gillespie algorithm for RNA kinetics with logarithmic time update. *Nuc Acids Res* 43(12):5708-5715.
8. Geis M, Flamm C, Wolfinger MT, Tanzer A, Hofacker IL, Middendorf M, Mandl C, Stadler PF, and Thurner C (2008) Folding kinetics of large RNAs. *J Mol Biol* 379(1):160-173.
9. Flamm C, Hofacker IL, Stadler PF, and Wolfinger MT (2002) Barrier trees of degenerate landscapes. *Zeitschrift für physikalische chemie* 216(2):155.
10. Voss B, Meyer C, and Giegerich R (2004) Evaluating the predictability of conformational switching in RNA. *Bioinformatics* 20(10):1573-1582.
11. Flamm C, Hofacker IL, Maurer-Stroh S, Stadler PF, and Zehl M (2001) Design of multistable RNA molecules. *RNA* 7(2):254-265.
12. Morgan S, Higgs P (1998) Barrier heights between ground states in a model of RNA secondary structure. *J Phys A: Math Gen* 31:3153-3170.
13. Dotu I, Lorenz WA, Van Hentenryck P, and Clote P (2009) Computing folding pathways between RNA secondary structures. *Nuc acids res* 38(5):1711–1722.
14. Gruber AR, Lorenz R, Bernhart SH, Neubock R, and Hofacker IL (2008) The Vienna Websuite. *Nucleic acids research* 36(2):W70–W74.
15. Gillespie, Daniel T (1991) Markov processes: an introduction for physical scientists. Elsevier
16. Kawasaki K (1966) Diffusion constants near the critical point for time-dependent Ising models. I. *Phys Rev* 145(1):224.
17. Senter E and Clote, P (2015) Fast, approximate kinetics of RNA folding. *J Comp Biol* 22(2):124-144.
18. Draper DE and von Hippel PH (1978) Nucleic Acid Binding Properties of *Escherichia coli* Ribosomal Protein S1. *J Mol Biol* 122:321-338.
19. Studer SM, Joseph S (2006) Unfolding the mRNA Secondary structure by the bacterial translation initiation complex. *Mol Cell* 22:105-115.
20. de Smit MH, van Duin J (2003) Translational standby sites: how ribosomes may deal with the rapid folding kinetics of mRNA. *J Mol Biol* 331:737-743.
21. Calander R (2006) The bacteriophages. Second Edition. Oxford University Press
22. Milon P, Maracci C, Filonava L, Gualerzi CO, Rodnina MV (2012) Real-time assembly landscape of bacterial 30S translation initiation complex. *Nat Struc Mol Biol* 19(6):609-615.
23. Rodnina MV (2018) Translation in Prokaryotes. *Cold Spring Harb Perspect Biol* doi:10.1101/cshperspect.a032664.
24. Salis HM, Mirsky EA, Voigt CA (2009) Automated design of synthetic ribosome binding sites to control protein expression. *Nat Biotech* 27(10):946-950.
25. de Smit MH, van Duin J (1990) Secondary structure of the ribosome binding site determines translational efficiency: A qualitative analysis. *Proc Nat Acad Sci* 87:7668-7672.
26. Calogero RA, Pon CL, Canonaco MA, and Gualerzi CO (1990) Selection of the mRNA translation initiation region by Escherichia coli ribosomes. *Proc Nat Acad Sci* 85:6427-6431.
27. Mathews DH, Sabina J, Zuker M, Turner DH (1999) Expanded sequence dependence of thermodynamic parameters improves prediction of RNA secondary structure *J Mol Biol* 288(5):911-940.
28. Dykeman EC, Stockley PG, Twarock R (2013) Packaging signals in two ssRNA viruses imply a conserved assembly mechanism and geometry of the packaged genome. *J Mol Biol* 425:3225-3249.
29. Lago H, Parrott AM, Moss T, Stonehouse NJ, Stockley PG (2001) Probing the kinetics of formation of the bacteriophage MS2 translational operator complex: identification of a protein conformer unable to bind RNA *J Mol Biol* 305(5):1131-1144.
