# Supplementary material for: Modelling ribosome kinetics and translational control on dynamic mRNA
Source: PLoS Comput Biol. 2023 Jan 23;19(1):e1010870. doi: 10.1371/journal.pcbi.1010870 (PMC9894550; doi:10.1371/journal.pcbi.1010870)
Supplement: Attachment — Submitted filename: response.pdf [file pcbi.1010870.s002.pdf]

Dear Colleagues,

I wish to thank both reviewers for their detailed reading of the manuscript and the extensive feedback that they have provided. I am grateful for the considerable time that they have both spent accessing the scientific merits of my work and providing me with such detailed feedback. My responses to the reviewers' questions and comments are highlighted in blue text below.

Reviewer #1: In this manuscript Eric Dykeman develops a computational model of co-translational folding of messenger RNAs and compares the predictions of the model to experimental data on translation of two translationally coupled proteins of the MS2 virus. The model appears novel and its results make it clear that the interplay of RNA structure kinetics, ribosome initiation, and ribosome motion can lead to unexpected consequences and thus has to be comprehensively modeled to obtain a quantitative understanding of these processes. Thus, this model is in principle valuable. However, the manuscript as written has many shortcomings, is difficult to follow, and leaves important questions open. It thus requires significant revisions before it can be reevaluated.

Major issues:
=============

1) There are many many bells and whistles in this model. On the other hand, there seems to be fairly little actual comparison to experimental data in this manuscript (see also point 2) below). It would thus be important to know, which of these details actually matter and which would allow other choices (or omission altogether) without degrading the comparison to experimental data. These are:
1a) The model considers ribosomes not only on the one structured RNA of interest but also on all other mRNAs in the cell (albeit treating them as without structure). Is this really necessary? It seems that this results in spending very significant computational resources on what probably mostly amounts to renormalized concentrations of available ribosomes and tRNAs.

Theoretically, yes one could re-normalize the ribosome 30S and 50S subunits along with the ternary complex concentrations and perform the protein synthesis calculation on just the mRNA of interest, in this case MS2 viral genome. When I was developing my original code (PMID 32049979) the original intent was to calculate protein synthesis on mRNA using detailed kinetic information and use re-normalised concentrations of 30S and 50S subunits and tRNA, using estimates for the levels of free 50S and 30S subunits in the cell, along with individual tRNA concentrations. However, upon computational experimentation, I have found that estimating these concentrations is not so straight-forward and they tend to be transcriptome dependant. Moreover, when you perform protein synthesis calculation on the full transcriptome (see PMID 32049979), you find additional paradoxes which are consistent with experimental observations, but have substantial consequences for the initiation process. One such example is that of the free concentrations of 30S subunits,

which are predicted to be substantially lower (about 4 fold) then free 50S subunits. In the quasi-steady state, a substantial amount of 30S subunits are predicted to be interacting with TIRs as a pre-initiation complex prior to 50S subunit joining. This results in 85% of ribosomes being in the elongations state (as observed/estimated experimentally) but 15% of the “free” 30S subunits are partitioned into truly free and TIR interacting. Thus, the modelling suggests that 30S and 50S subunits balance in a substantially different ratio in the cell as a consequence of the two stage initiation process (30S joins as a PIC followed by 50S joining after identification of start codon). If this true or not in reality would require additional experimental confirmation, but it has highlighted for me the difficulties of estimating free concentrations of 30S and 50S subunits and tRNAs in the cell. For all these reasons, I thought it would be a better estimate of the protein synthesis behaviour of the MS2 mRNA if the competition from the background host cell mRNAs was present. Although this appears to be a substantial computational cost to account for in the simulation, it is only minimal. This is due to the Log(n) Gillespie method that I use, where adding more mRNAs to the simulation increases computational costs roughly logarithmically as discussed in the text.

1b) Why are overlapping TIRs pruned to just the one with the lowest interaction free energy? Aren't all TIRs in principle available to the 30S subunit to bind to?

The ability of the ribosome to initiate anywhere on the mRNA still has some issues and needs some additional corrections to the model which is an on-going process. One of these issues is that, if you include all possible TIRs, then the ribosome starts initiating practically everywhere (mainly on alternative start sites around the correct start codon) with only a handful of correct starts on the actual gene. In MS2, there are two alternative AUG and GUG start codons near the correct start codon. These two alternative starts are out of frame, overlap with the correct start site, and together out compete the main start. Pruning fixes this issue by essentially assuming that, the 30S subunit will have time to thermodynamically equilibrate on the lowest energy TIR in the region. This makes some sense as the 30S joins as a stand-by site waiting for hairpin melting. However, I admit it is a bit of a fudge for the moment until more experimental evidence comes about on the actual steps of 30S interaction with the TIR. I have added a discussion about this issue in the text highlighting that there are still issues like these with the ability of the 30S subunit to initiate anywhere. I have also added to the code the ability of users to explicitly limit initiation to only specific sites.

1c) Is actually anything beyond the hairpin containing the start codon relevant (see also 2b))

The answer to this question is also addressed in the answer to 2b.

1d) There seem to be a lot of parameters going into the description of 30S binding. Are the results sensitive to all of these?

There are essentially two main parameters for 30S binding, TIR melting time (tau) and the binding rate of 30S to mRNA via S1 protein (k1). There is the additional parameter related to 30S interaction energy with the mRNA, but this mostly controls the off rate as opposed to the 30S binding rate. There are 6 additional parameters for 50S subunit joining which will govern quality control for initiation at the start codon. Some of these parameters slow drastically for AUU codons etc. which could in the future be incorporated for a quantitative estimate of initiation at non-AUG codons. However currently there is limited kinetic measurements of how these kinetic rates change for non-AUG codons. I have added a comment in the text about initiation at non-AUG or GUG codons lacking accuracy at this stage due to these missing measurements. Regarding 30S binding, it is just k1 and tau that the relative protein ratios are sensitive to.

2) Validation of the model by comparing it with experimental data is of course key for any modeling paper. However, there are various issues with this validation (see also point 9)):

2a) What is actually compared to experiment? It seems that the general form of equation (2) is independent of all the model details. So, is the only thing that actually comes out of running this entire highly complex model the values of the parameters A and B? This seems to be very little information to claim validation based on given all the many parameters that go into the model.

Actually, A and B are dependent on all of the conditions for the cell, another reason why I have done the simulation with background mRNAs present. If one alters features such as the amount of cellular mRNAs present, tRNA concentration, or growth rate, then the relative expression (and corresponding A and B values) will change as a result. The settings in the simulation (temperature and estimated growth rate) match those done in the van Duin experiments. Thus, it is incorrect to say that Equation 2 is independent of all of the model details. This was one of the reasons for reporting on the resulting predicted folding times for the Hairpin on lines 327-347 as the folding times resulting from the protein expression fit are very close to independent estimates (Porsche and van Duin) and theoretical (KFOLD) folding times for the RNA hairpin.

Regarding comparison of protein expression to experiments. The main issue with translational coupling experiments in MS2 is that, only the relative ratios of coat protein synthesis have been measured via western blotting. There is currently not a measurement of a ratio of RdRP/Coat. I suspect that the main reasons why are that RdRp is produced in small amounts in the cell and that RdRp is toxic to E. coli.

What is needed is additional experiments which take the MS2 coat gene and attempt to measure the coupled gene somehow. Currently I am working on precisely this. I have designed a synthetic mRNA where RdRp has been replaced with a fluorescent reporter gene, circumventing the toxic RdRp issue and allowing a direct measurement of the downstream coupled gene resulting from different levels of coat protein synthesis. These

experiments are on-going, but nearing completion. The issue was either to wait for these experiments or report on the model by itself with some experimental support from the van Duin measurements. I felt that the computational advances in the model warranted a separate publication.

2b) Do lines 340-347 really say that the only thing that really matters for this comparison with the experiment is the unfolding time of the coat hairpin and that KFOLD actually gets this parameter mostly right? If that is true, it does not seem as if this comparison to experiment really provides a validation of the model as a whole.

The reason for this comparison is twofold. The first reason is that, as noted in the answer to 2a above, by fitting A and B one obtains an estimate for the coat Hairpin folding time. Since A and B are dependent on the translational dynamics of the coat gene, any inaccuracies in the translational simulation will translate into errors in the implied hairpin folding time which comes out of the fit to experimental data. This provides an opportunity for a “sanity” check, as this value should coincide with an independent theoretical estimate from RNA folding kinetics calculation.

The second reason is that previously I had a referee on an RNA folding paper of mine who questioned if one could calculate the hairpin melting times from a mean-first-passage time calculation using a breadth first search algorithm that identified a single “best” RNA unfolding path (see SI document). I included the KFOLD comparison in this paper (supplementary Table S3 and main Table 1) to show that indeed when compared to an “exact” calculation of the average melting time using KFOLD which simulated unfolding at single-base pair steps, the unfolding time computed from the breadth first search algorithm is approximately the same, within a small factor < 2.

2c) Figure 5B is actually not very useful for assessing the quality of the agreement between the experimental data (red dots) and the theoretical line. The two data points at high relative protein expression (which the author tells the reader to ignore in the comparison) "squeeze" the actually important data points into a narrow range of y values that makes deviations appear very small. Indeed, e.g., mutant 22 is off by a factor of 2 according to table 1, which is of course not visible in figure 5B since both, the measured and the theoretical value are small. Plotting the measured and theoretical values from table 1 against each other, preferably on logarithmic axes would provide a more honest comparison.

This is a fair point. Originally, I had thought that table together with the figure would be transparent enough. But I agree the figure may give the wrong impression. I have replaced it with a Log/Log plot in Figure 5.

3) The paragraph in lines 239-252 says that it is possible to parametrize the model such that the range of off rates agrees with the range of experimentally measured off rates. However, the ranges themselves cover many orders of magnitude. Is there agreement with the experimentally measured off rates on a sequence by sequence level (which would

be a much stronger statement) or is the agreement really only the general range?

The large range of off-rates comes from experimental fluorescently labelled binding measurements that were performed by Joseph and Studer in Ref 18. The variety of sequences that they examined were somewhat limited. The Shine-Dalgarno sequences examined were either (1) a poly-U sequence or (2) the full-length SD complimentary to the 16S ribosome RNA. So essentially, they ended up with two measurements which gave the range quoted, one for the full-length sequence which gave off rates ~ 10^-4 per second and the poly-u sequence which had off rates > 1 per second, depending on which Initiation factor subunits were present. I have parameterised the model so that the off rates lie between these two measurements (by order of magnitude).

4) The author's argument for why a coarse grained approach of the RNA kinetics is appropriate is that the time scale of ribosome movement is a slow 50ms, which is thus slower than (most) RNA folding kinetics. While this is true, another important aspect pointed out in the abstract is that the author includes protein RNA interactions. Wouldn't those be on faster time scales and thus might not be well described by this coarse grained approach?

An excellent point. Here I would point out the following estimate which shows that protein binding is also relatively slower then RNA folding. First is that the most abundant protein in the cell is EF-Tu, which at high bacterial growth rates will have concentrations in the range of 0.1 - 0.2 mM. Assuming kinetic rates of binding of Ef-Tu in the range of 10^6 per M per sec, which is nearly diffusion limited rate for the protein, then one can expect the time it takes for Ef-Tu protein to bind to the ribosome is 5-10 ms. This makes sense with the overall elongation rate (5-10 ms of the 50ms is due to Ef-Tu recruitment to the ribosome as Ternary Complex). Other cellular proteins in the cell will not reach this level of concentration, so should take longer to bind to mRNAs or other proteins. Estimates for the number of viruses assembled in MS2 range from 100-1000, which gives a range of final coat protein concentration in the cell of 10uM to 100uM. The latter estimate is near the concertation of Ef-Tu in the cell, so at worst one could estimate that coat protein to mRNA and protein binding to mRNA in general (viral or host cell proteins) is no faster than roughly 5ms. (I have added these estimates to supplementary discussion). While a factor of 10 faster than ribosome translocation, this is still longer than most small local hairpin folding reactions (< 1ms). Moreover, the lifetime of the protein bound to the mRNA also needs to be accounted for. Specifically, a protein could bind mRNA, dissociate, then the mRNA re-fold sequestering the binding site of the protein. All these details are accounted for in the coarse-grained model. Essentially the main issue with the coarse-grained model will be the reduction of mRNA folding states instead of the folding time issue (see response to point 6 below), as the effects of time differences between RNA folding and protein binding and ribosome translocation are accounted for.

5) In line 73 the author says that the model treats the RNA folding "at the single nucleotide level". Yet, later it becomes clear that the RNA folding is treated in a coarse grained way. The author should weaken the claim in the introduction accordingly, so as to not confuse the casual reader.

I see that this wording is confusing. What I wanted to emphasise is that each nucleotide of the sequence is present, and so the actual RNA fold is not abstracted into “helices” as is done for the tree representation. Moreover, the coarse-grained model does calculate pathway transitions at the single nucleotide level using a breadth first-search algorithm. So, the “coarse-grained” RNA folding in the model here is only from the reduction in mRNA state space. I have clarified that coarse-grained in this work means reduction in RNA state space in the manuscript text.

6) In line 272 it is stated that the input to the model is the sequence and the structure of the RNA. It is somewhat surprising that the structure is necessary, and it is not very clear in how far this provided structure constrains the potential structures the RNA visits during the simulations. Would alternative structures that are not subsets of the provided structure be forbidden? Probably not, since the methods do talk about refolding subsequences of the RNA. But the set of alternative structures is somehow limited by the input structure, isn't it? The manuscript should spell out crystal clear in which way the set of possible structures visited during the simulation is limited by the input structure. This seems to be a key point of the whole method (and a possible weakness in cases of RNAs with multiple alternative structures).

The structure is necessary because we need an ***initial*** structure that the ribosome will “see” prior to interacting with it. This is relevant for viral mRNAs as they enter the cell already folded. This may be computed either from a co-tran**scriptional** folding algorithm, or preferably with input from experiment. With MS2, we are fortunate to have well studied RNA structure probed using gold-standard techniques (enzymatic probing with co-variation analysis). Regarding how much this structure constrains which structures it visits, the model does not restrict the alternative structures of the mRNA per-se since, as you have noted, there is a calculation of alternative structures in different “windows” or different sub-sequences of the mRNA. However, there will be some restriction due to the coarse-graining of the RNA state space. Theoretically, the method of Geis et all (Ref 8 in SI) on which my method is based, should allow the RNA to re-fold any subsequence to produce a locally optimal fold (lowest energy fold on this subsequence). Thus, the mRNA state space here considers approximately all mRNA folds constructed from locally optimal folds. One way to increase the number of states that the mRNA has access to is to consider many possible local folds, not just the lowest energy. This is potentially feasible computationally, and I have some ideas on an algorithm, but currently such an algorithm does not exist. I have added text around line 72 highlighting these issues and limitations of the current algorithm.

7) The finding of translational coupling between the coat protein and RdRp is quite interesting. However, it seems that the comparison to experiment here is purely qualitative. Is there a way to make more quantitative statements? After all, the abstract says that the model "accurately reproduces" this behavior. What does "accurately" mean if no numbers are compared between experiment and modeling? Can it at least be made clear which of the findings are predictions for (potential) future experiments and which are actually compared (favorably) to existing experiments? This entire section is also somewhat difficult to understand. It should spell out explicitly what type of protein binding is included. Is every hairpin a potential binding site for a coat protein? Or just one? And how was figure 6B obtained (specifically what was actually varied to obtain the different hairpin unfolding times? Some model parameter? The hairpin sequence?)

Some of the answer to this question is addressed in point 2a above. At the moment, I would agree that the model is qualitative as it reproduces the coupling and repression behaviours that is expected, but the quantitative predictions on the amount of RdRp relative to Coat requires additional experiments that have not been performed (but I am in the process of completing – see response to point 2a above). I have added a section in the discussion detailing this issue and highlighting that the model predicts relative coat protein expression, but the amount of RdRp requires further experimental measurements.

Regarding the protein binding, the protein is allowed to bind to any potential hairpin site, not just TR hairpin. This is critical since MS2 has multiple coat protein binding sites which are involved in assembly. This has been added to the manuscript.

Finally, to obtain figure 6B, the value of tau is varied directly for creation of the plot. However, according to the mutants listed in Ref 11, one can add just the sequence and let the code predict the melting time based on the sequence data and the Hairpin Delta G values. I have added this to the figure legend.

8) Could the author comment on the discrepancies observed in table S3, especially the one for mutant 11 at 37 oC? Does this factor of 3 matter?

Essentially the pathway between hairpin and open structure calculated from KFOLD allows the RNA to transition over many different trajectories between the two states. Some of these may be indirect pathways, where base-pairs not involved with the original structure form temporarily during Hairpin melting. The pathway calculated from the breadth first search algorithm is direct, in that it does not consider these in-direct pathways that involve other base-pairs do not present in the structure. Naturally one possibility is that there is an in-direct pathway that is skewing the results. There are algorithms which account for this issue, however these are more complex and require larger amounts of computational time.

Another possibility is that due to the long melting time (estimated 13 sec in breadth-first search) there may not be enough sampling to fully sample pathways which take a long time (i.e. these are underrepresented). KFOLD was taking several weeks to perform the melting simulations, suggesting some predicted times are very large. There is an upper

limit on the folding time which was set to 15 sec to reduce the computational time. Thus, if there are a substantial number of pathways above this cut-off, this would skew the results. This is probably what has happened in all likely hood. Discussion has been added to supplementary.

Minor issues:
=============
9) Something went really wrong with the paragraph presenting the comparison with the experiment (below equation (4)). All the figure references here are to figure 3. At least most of them should probably be to Figure 5 instead? But maybe not the first one? And why are the panels in figure 5 in the order in which they are? Shouldn't D precede B logically? That also seems the order in which they appear in the text. This is really the key section of the manuscript since it is the comparison to the experimental data that is supposed to validate the model and confusing the reader here by sloppy figure references is really just a bad idea.

Many thanks for noticing this error. It seems the links in the Latex file where incorrect, so the references to the figure numbers were off. I have fixed these. I have also re-positioned the figures so that the experimental data comes first, followed by the modelling data and fit (with log/log plot), which I agree is a more natural progression.

10) The discussion around equation (1) is very difficult to follow, especially without first reading the supplemental material. Please provide enough context that the logic behind what is presented here becomes clear without knowing all the methodological details already.
I have added additional text around Eq 1 and its derivation in the main manuscript.
11) Another difficult to read section is the discussion of the translational results in lines 394-402. Talking about "plateauing" is counter-intuitive if said "plateauing" is only seen when reading figure 6B from right to left and it is also left to the reader to make the connection that "weaker" hairpin means shorter unfolding times (which is the x axis on figure 6B).

After re-reading this section I see that it is indeed not clear. I have adjusted the text appropriately.

12) It is a strange choice to leave essentially the entire description of the algorithm to the supplement and have just one paragraph of methods in the main text. After all, the whole manuscript is primarily about the model. And there is no length restriction in this journal, is there? The author should consider moving some of the supplemental material from the supplement into the main text. While doing so, it would also be helpful to be more explicit about what the allowed steps in the Gillespie algorithm are, which is somewhat implicit in this current version.

Thanks for these comments. While the supplementary material contains sufficient information on the algorithms such that another person could implement them, I felt that

due to the technical nature of the material and overall length (>17 pages), it was best left for supplementary while the main manuscript demonstrated the program on the example of MS2. When you ask about the allowed steps in the Gillespie algorithm, I assume that you are asking about what the allowed reaction steps are. Most of this information is in my original ribosome paper (PMID 32049979). However, it would be good to re-mention at least the overall set of reactions that are considered. I have added text around the discussion of Figure 1 so that the full extent of the reactions is understood by the reader.

13) The author should try to be cognizant of significant digits. Is the temperature quoted in line 329 to five significant digits really controlled to that precision in the experiments that are referred to? Is the intercept in line 330 really reliable to 5 significant digits?

Thanks for pointing this out. I have adjusted beta to the appropriate number of significant figures.

14) The choice of using the capital letter K for rates seems non-standard. Typically, capital K stands for equilibrium constants while rates are denoted by lower case k.

I have switched all upper case K (where they are rates) to lower case k to be consistent with convention.

15) There are a lot of typographical errors in the manuscript, which make the entire work appear very sloppy. It should be standard practice to at least spell check a manuscript before submission:
- abstract: "co-translational fold" -> "co-translationally fold"
- line 23: "dependant" -> "dependent"
- line 36: "their" -> "there"
- line 37: "uses" -> "use"
- line 38: "its" -> "their"
- line 148: "to computed" -> "to be computed"
- line 210: "week" -> "weak"
- line 214: "specifcially" -> "specifically"
- line 240: "then" -> "than"
- line 268: "imputing" -> "inputting"
- line 298: "42 degrees C" -> turn "degrees" into degrees symbol
- line 299: "42 C" -> add degrees symbol
- line 303: "results" -> "result"
- line 307: "Shine-Delgarmo" -> "Shine-Dalgarno"
- line 362: "snapshpot" -> "snapshot"
- line 367: "effected" -> "affected"
- line 399: "platues" -> "plateaus"
- line 401: "platue" -> "plateau"
- Table 1: add index "F" to Delta G
- Supplemental material page 1: "M61655" -> "MG1655"
- Supplemental material page 3: "structure needs accounts" -> "structure accounts"

- Supplemental material page 12: "16s" -> "16S"
- Supplemental material page 12: "M" -> "mol" (molar and mole are different!)

Many thanks for your careful reading of the manuscript and identification of these typos. I have made the appropriate revisions in the manuscript and done a final spell check on a separate program as my tex program seems to be malfunctioning on the spell check here.

Reviewer #2: Review of PCOMPBIOL-D-22-01546

Dr. Dykeman developed an algorithm for the simulation of protein synthesis rates in E. coli. His approach is unique in the sense that he combines a very detailed model of mRNA translation with mRNA folding dynamics to assess translation initiation rates.
Overall, the manuscript is well written and has a clear structure. The results are well presented and the reader can understand how they were obtained. The presented work is a very nice achievement and its applicability might only be hampered by the lack of experimental data on in-vivo mRNA secondary structure (which should not be of relevance for assessing a computational model). I recommend publication after the following concerns have been addressed.

Major concerns:
1) The author needs to put his work in a broader context of what has been achieved before. There is a thorough discussion only of refs. 11 and 26 (if I did not miss anything). But a quick internet search reveals for example the paper PMID: 20504310 that also claims to introduce a “model [that] uses mRNA-folding dynamics and ribosome-binding dynamics to estimate translational efficiencies solely from mRNA sequence information”. The author should inform the reader of more approaches alternative to his and give a hint how these approaches differ from the presented one.

Many thanks for this comment. I agree that it is important to put this work into context with previous work in the field. The main incorporation of RNA structure into protein translation models have come from examining the role of mRNA structure on initiation. Indeed, there are several well thought out models on this (Salis, in ref 13 and 26 and the PMID that you have identified which is essentially the same concept as ref 13). The difference here is in the monitoring of the whole mRNA secondary structure (not just structures in the TIR) and its effects on (1) translational coupling and (2) translational repression. Outside of work done on mRNA structure in the TIR, I am not aware of other groups looking at structure of the mRNA in other areas and its effects on translation. I have added your identified reference and amended some text to the manuscript to point out the difference with the work here and previous models.

2) The same concern applies to mathematical models and simulations of mRNA translation in general. I understood that the author here presents a further development of his previous algorithm. Nevertheless, it is necessary (and informative for the reader) that he briefly compares (or advertises) his underlying model for computation of translation

rates to that of other researchers. A detailed mechanistic model has been applied for example in PMID: 31101858 and PMID: 31369559, and there are a few more examples in the literature of detailed models and more simple measures (like tAI or CAI).

Reviewer 1 has also pointed out that additional details on the reaction's steps would be useful to be restated in this paper. I have added this info, along with the references listed in the above comment as they are useful to put the translational model here in context with similar excellent work by others in the field.

3) Further citation is needed, for example on lines 31/32 (statement), 36-39 (statement), 290/291 (parameter values), caption of Table 1 (to Turner 99). The author should check if he missed further citations.

I have added reference links to appropriate papers on lines 31/32 and 36/39 and 290/291. I have added the missing reference in Table 1 as well.

4) Code availability: Currently, the software is available from the author's homepage and the FORTRAN code is accessible after download. For a better general accessibility, findability, reusability and visibility (among others) it would be favorable to host the code on a public repository (such as Gitlab, Github). This would also enable direct interaction with users via creation of issues or even further development of the code by the community. (The author might even think about developing a SBML model to be published at the BioModels database if that is possible.) See also journal's statement: "The data and code should be provided as part of the manuscript or its supporting information, or deposited to a _public_ repository".

I am in the process of developing a more user-friendly version of the code that is planned to be deployed on a repository such as Github, along with tools for creation of parameter data such as tRNA concentration profiles, etc that can be imported into the code for testing and simulation. Until then, I thought it would be best to offer the current software on my own website, so that when the more user-friendly version goes live on the Github repository, users can be more easily re-directed to the correct newer software.

I have a few more questions and would be glad if the author could comment and/or adjust the text accordingly:
1) Are the predicted TIRs confirmed by known start-codon positions?

The algorithm identifies all the correct start codons known in bacteriophage MS2, along with some additional sites which are not functional as genes, but may elicit some very rare translational starts. If these additional sites do result in some translational starts would be of interest to know, however it requires some experimental work.

2) Line 201: Why these codons (as alternative start codons) but not AUU?

There is an experimental paper which looks at translational starts at all 64 codons, and

finds that the codons with high probability are AUG followed by GUG. There is some smaller chance to initiate at other start codons 1 mutation away from AUG. These are UUG, CUG, AUC AUA and AUU. AUU was omitted by accident. The idea in the code was to allow starts at these sites, but to obtain the correct initiation rate, we need to know how these codons effect 50S subunit recruitment, part of Rodina and colleagues “checkpoint 4”. I have added additional text in the manuscript to clarify this, and that the model is not yet accurate for these alternative start codons without additional experimental measurements.

3) Line 206: Why are TIRs removed from the list based on \delta G, i.e., “binding strength” of the 30S:PIC? Is there experimental evidence that TIRs are optimized in that sense?

I found that, by including all possible start sites, most initiations occurred at alternative overlapping TIR sites. There are two additional AUG codons which overlap the authentic coat AUG. Although these have higher Delta G of interaction with the 30S subunit, together they seem to out compete the authentic site. So, by removing the additional overlapping sites thorough a lowest energy argument, this fixes the issue, albeit in a slight “fudge”. A simple justification would be that since 30S is recruited as a stand-by, the 30S has time to sample TIRs and reaches thermodynamic equilibrium on the lowest energy site. This was also pointed out by the other reviewer and text has been added in the manuscript discussing this.

4) Line 299: Where rates also corrected for temperature or are the differences for T = 42°C vs. 37°C considered to be too minor?

I am unaware of estimates available for adjusting the kinetic rates of the ribosome due to temperature changes. Although It is fairly reasonable to expect that, since E coli grows fairly well at 42, that the ribosome kinetics is mostly unaltered. I have added a brief comment in the manuscript about this issue.

5) Eq. 2): Could you please elaborate? What are the meanings (not just definitions) of the constants A, B, C?
I have added additional text in the main manuscript regarding the derivation of Eq. 1. It is there that I have added some additional comments regarding the meaning of A,B, and C.

Further minor issues:
1) The manuscript could be written more concisely. See for example repetitions in line 268 (p. 7) vs. line 272 (p. 8) or line 282 (p. 8) vs. 284 (p. 8).
2) The manuscript needs thorough typo correction, for example lines 327-338 refer to Fig. 3 instead of Fig. 5.
3) Line 193 “appearing and disappearing”: Is the TIR “disappearing” or just nor accessible?
4) Lines 249 and 251: The ranges [0.001, 10.0] and [0.001, 4] differ; in which sense is this “consistent”?

5) Line 139: The term “co-translational folding” is used differently (depending on context; referring to folding of the nascent chain). To avoid confusion, I suggest to specify the term, e.g., “co-translational mRNA folding”
6) Line 326: I would use “maximum” instead of “peak”.
7) Line 337, “excellent fit”: Did ref. 11 report error bars (especially concerning #18 and WT)?

Many thanks for your careful reading of the manuscript to identify these typos and language issues. I have made the appropriate changes in the manuscript.
