# Supplementary material for: Modelling ribosome kinetics and translational control on dynamic mRNA
Source: PLoS Comput Biol. 2023 Jan 23;19(1):e1010870. doi: 10.1371/journal.pcbi.1010870 (PMC9894550; doi:10.1371/journal.pcbi.1010870)
Supplement: Attachment — Submitted filename: response2.pdf [file pcbi.1010870.s003.pdf]

Dear Colleagues,

Many thanks again for your comments and careful reading of the manuscript. I have added changes to the manuscript, this time highlighted in blue text, along with the first revisions which remain highlighted in red. My responses to your individual comments are below in blue.

Reviewer #1: The author has taken into account most of the reviewers' criticisms in this revision and the manuscript has thus improved. It tells the story quite well now, but there are still a few remaining points:

1) The author provides an explanation of why it makes sense to model translation on all mRNAs rather than only on the mRNA of interest in the reply to the reviewers. Please add some of this useful explanation to the actual manuscript as well so that readers can benefit from it.

I have added some text to the manuscript discussing the benefits of modelling translation on all of the mRNAs that the reviewer requests. See the first paragraph of the Results section which discusses several advantages with an example of estimating free concentrations.

2) This reviewer is still not convinced by the author's answer to the original point 2b). The question was about what the comparison to the experiment is really probing. The manuscript showed that running KFOLD on the TIR hairpin essentially gives the correct results. Yet, the author's method looks at the folding of the entire mRNA. This reviewer's concern was that the fact that KFOLD gives more or less the right answer implies that *none* of the folding of the rest of the mRNA matters for the comparison to the experiment. Thus, the quantitative agreement with the experiment does not really test anything about the author's algorithm other than the implementation of the folding of the TIR hairpin. If that is so, the author must acknowledge that in the discussion.

I attempted to address this issue in the last iteration (see points 2a and 2b) but I see that further clarification is needed. The Van Duin experiments looked at only alterations to the coat hairpin. Thus, by their nature these experiments only probe the local effects of the secondary structure on the expression of the coat protein. This is not to say that more global effects with the secondary structure do not exist. But no experiments have been done to look at the effects of the global fold on coat expression. Thus, the folding of the global structure is not needed to compare to the experimental predictions of Van Duin et al. as you have noted.

The folding of the rest of the mRNA is, however, important for the prediction of the expression of the RdRp gene, as this is coupled to coat protein and the translation of RdRp is repressed by binding of coat protein to secondary structures in the RdRp gene (TR hairpin). The model reproduces this experimentally observed coupling and translational repression, but these comparisons are qualitative in nature, as experiments have not performed any quantitative measurements such as Coat/RdRp ratio, or simply a time profile of RdRp expression. The model does make predictions about the expression ratio (Figure 6 in main text) which will depend on the global secondary structure of the coat gene as well as the RdRp TIR. But as I mentioned previously, new experiments are currently being undertaken to measure and

corroborate the models' findings. I attempted a brief sentence in the first paragraph of the discussion to explain this. I have extended this to make these issues clearer to the reader.

3) The explanation of the comparison to the Joseph and Studer results is also not satisfactory. Is the author saying that Joseph and Studer did not really observe a range, but rather did two experiments, one with a Shine-Dalgarno sequence and one without a Shine-Dalgarno sequence and that the "range" really just represents these two points. If so, the manuscript should say that explicitly and instead of claiming that the author's method matches the range say that it matches the two measurements, the one with and the one without a Shine-Dalgarno sequence.

Joseph and Studer did several measurements of 30S off rates for small RNA fragments. However, only two different SD sequences were used, the full length SD and a poly U sequence (i.e. no SD). The other variants that the tested were looking at the impact of secondary structure on the off rates. I have added a comment in the manuscript which says that only the Joseph and Studer measurements only contribute two data points to the off rate curve.

4) It is quite disappointing that in spite of being an expert in modeling prokaryotic translation, the author consistently misspells Shine-Dalgarno in the entire manuscript!

I see that this issue has annoyed you. What happened was I did an auto-replace and swapped a for e by accident. I should have double checked. Sorry that you had to bring this issue up again and I appreciate your time checking the manuscript. However, being the editor for another journal, I am surprised by the tone of your response which is not in keeping with collegial format of Author/Reviewer correspondence that the journals, reviewers, and authors are supposed to adhere to.

5) Based on the response to the reviewers, please add "initial" in front of "secondary structure" in lines 307 and 311.

This change has been done.

6) The author claims to have replaced all the upper case Ks used for rates by lower case ks. However, many of the captions to supplemental figures still contain upper case Ks for rates.

Many thanks for double checking this. I had looked through twice during the first revision to make sure that all of the uppercase Ks were found, but sometimes they start blending together in the brain. I have also had a colleague look as well. So hopefully between three people they have all been identified now.

7) "then" -> "than" in line 462

This has been fixed.

Reviewer #2: My concerns were almost fully addressed, I have just two minor comments left:

1) I strongly encourage the author to publish the code on (e.g.) Github and state the link to this page in the paper (instead of the private one). Private homepages tend to disappear at some point and the reader has no chance to use the published code anymore. Also, there is no need to wait until the code is “ready” before publication on Github. In contrast, these repositories are meant for code “under construction” (version control with git etc).

I have made a github page for the code and added a link to the repository in the text.

2) “I am unaware of estimates available for adjusting the kinetic rates of the ribosome due to temperature changes”: A simple Arrhenius approach is probably sufficient to estimate rate differences between 37 and 42 degrees, similar to what has been done in PMID 25358034. Would that work in your case?

Thank you for pointing this potential way to obtain estimates for the ribosome kinetic rates at 42 C. In the paper you quote, the authors scaled the kinetic rates measured at 20 C to obtain kinetic rates at 37 C. To do this, a reference measurement of one of the kinetic rates (omega_45 in the paper) was needed at both 20 C and 37 C. To do a similar procedure here, a reference measurement of one of the kinetic rates would be needed at 42. I had a very quick look in the literature to see if I could find such a measurement, but I did not. I do know of several measurements in the literature in temperatures ranging between 20-37 (which are mostly discussed in PMID 25358034) but I don’t recall encountering any measurements at 42 C. I have added an additional line in the manuscript discussing the possibility of scaling the kinetic rates using the formula in PMID 25358034 given a reference measurement at 42.
